# A new serotonin 5-HT_6_ receptor antagonist with procognitive activity – Importance of a halogen bond interaction to stabilize the binding

**DOI:** 10.1038/srep41293

**Published:** 2017-01-24

**Authors:** Juan A. González-Vera, Rocío A. Medina, Mar Martín-Fontecha, Angel Gonzalez, Tania de la Fuente, Henar Vázquez-Villa, Javier García-Cárceles, Joaquín Botta, Peter J. McCormick, Bellinda Benhamú, Leonardo Pardo, María L. López-Rodríguez

**Affiliations:** 1 Departamento de Química Orgánica I, Facultad de Ciencias Químicas, Universidad Complutense de Madrid, E-28040 Madrid, Spain; 2 Laboratori de Medicina Computacional, Unitat de Bioestadística, Facultat de Medicina, Universitat Autònoma de Barcelona, E-08193 Bellaterra, Spain; 3 School of Pharmacy, University of East Anglia, NR4 7TJ Norwich, UK

## Abstract

Serotonin 5-HT_6_ receptor has been proposed as a promising therapeutic target for cognition enhancement though the development of new antagonists is still needed to validate these molecules as a drug class for the treatment of Alzheimer’s disease and other pathologies associated with memory deficiency. As part of our efforts to target the 5-HT_6_ receptor, new benzimidazole-based compounds have been designed and synthesized. Site-directed mutagenesis and homology models show the importance of a halogen bond interaction between a chlorine atom of the new class of 5-HT_6_ receptor antagonists identified herein and a backbone carbonyl group in transmembrane domain 4. *In vitro* pharmacological characterization of 5-HT_6_ receptor antagonist **7** indicates high affinity and selectivity over a panel of receptors including 5-HT_2B_ subtype and hERG channel, which suggests no major cardiac issues. Compound **7** exhibited *in vivo* procognitive activity (1 mg/kg, ip) in the novel object recognition task as a model of memory deficit.

The serotonin 5-HT_6_ receptor (5-HT_6_R) belongs to the important G-protein coupled receptor (GPCR) superfamily of drug targets^1^. Extensive investigation has shown that 5-HT_6_Rs regulate several neurotransmitter pathways including at least serotonergic, cholinergic, glutamatergic, and GABAergic systems^2^. Interestingly, today there is compelling evidence that the receptor is involved in learning and memory processes and the ability of 5-HT_6_R antagonists to improve cognitive function has been well established at the preclinical level^3^,^4^. Several of them have been consistently shown to significantly enhance the memory retention or formation in rodents in multiple behavioural models reflecting diverse cognitive tasks. In addition, these antagonists have been demonstrated to reverse pharmacologically induced cognitive deficits in a number of cognition paradigms. Further substantiation has been provided from emerging clinical data. Indeed, several 5-HT_6_R antagonists have advanced to clinical development, and to date, at least three of them have reached phase II/III trials as drug candidates for cognitive enhancement, which holds promise for these compounds as novel therapeutic agents for the treatment of Alzheimer’s disease (AD)^5^. This neurodegenerative disease is one of the greatest human health challenges in this century because of its clinical and socioeconomic impact worldwide. Despite currently available medicines, there is still a clear need to develop novel therapeutic approaches with improved overall clinical benefit to alleviate the cognitive symptoms of the disease. Therefore, serotonin 5-HT_6_R has become an attractive drug target for cognition enhancement in AD and other diseases where memory loss and learning complications are symptoms^5^,^6^,^7^. Nevertheless, the development of new 5-HT_6_R antagonists is still needed to validate these molecules as a drug class for the treatment of AD.

Structure-activity relationship studies as well as complementary methodologies of drug design, such as pharmacophore-based and structure-based homology models, have allowed the identification of four common key structural elements in 5-HT_6_R antagonists (Fig. 1A): a positive ionisable atom (PI, in red), an aromatic ring (AR, in yellow), a hydrogen bond acceptor group (HBA, in green), and a hydrophobic site (HYD, in blue)^8^. The AR region is occupied by a mono- or bisaryl π-electron donor aromatic or heteroaromatic system (indole ring in most cases) that is used as central core, where the remaining pharmacophore features are accommodated in an appropriate spatial orientation^5^. As part of our efforts to develop 5-HT_6_R antagonists, we have previously contributed with a new family of compounds based on a benzimidazole ring as central AR core. Representative ligand **1** (*K*_i_ = 34 nM) contains 4-methylpiperazine moiety as PI pharmacophore element, a carbonyl group as HBA, and a naphthalene ring as HYD (Fig. 1B)^9^. In the present work we further explore the benzimidazole system as a central scaffold to identify 5-HT_6_R antagonists. In the new series the key structural elements are anchored to the benzimidazole ring as depicted in Fig. 1C. Although structural information of the 5-HT_6_R is presently unavailable, the recently provided crystallographic data for the serotonin 5-HT_1B_ and 5-HT_2B_ receptors^10^,^11^ and mutagenesis studies have been used to develop molecular models of the 5-HT_6_R in complex with the synthesized compounds, which have shown the importance of a halogen bond interaction in the HYD element to stabilize the binding. Analogue **7** (*K*_i_ = 9 nM) containing (dimethylamino)ethyl fragment as PI, a sulfonamide group as HBA, and 5-chloro-2-naphthyl as HYD (Fig. 1D) has been characterized as a selective 5-HT_6_R antagonist that exhibits no important pharmacokinetic issues and *in vivo* procognitive activity in the novel object recognition task.

## Results

**Figure 1:**
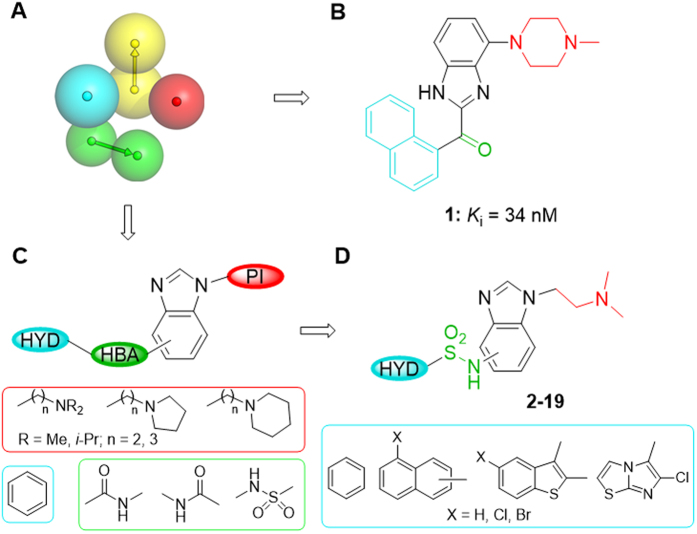
Benzimidazole-based compounds targeting the serotonin 5-HT_6_R receptor. (**A**) Pharmacophore elements for antagonists are a positive ionisable atom (PI, in red), an aromatic ring (AR, in yellow), a hydrogen bond acceptor group (HBA, in green), and a hydrophobic site (HYD, in blue). (**B**) Previously reported antagonist with benzimidazole ring as central AR core. (**C**,**D**) New series of compounds with the key structural elements anchored to the benzimidazole ring.

**Figure 2:**
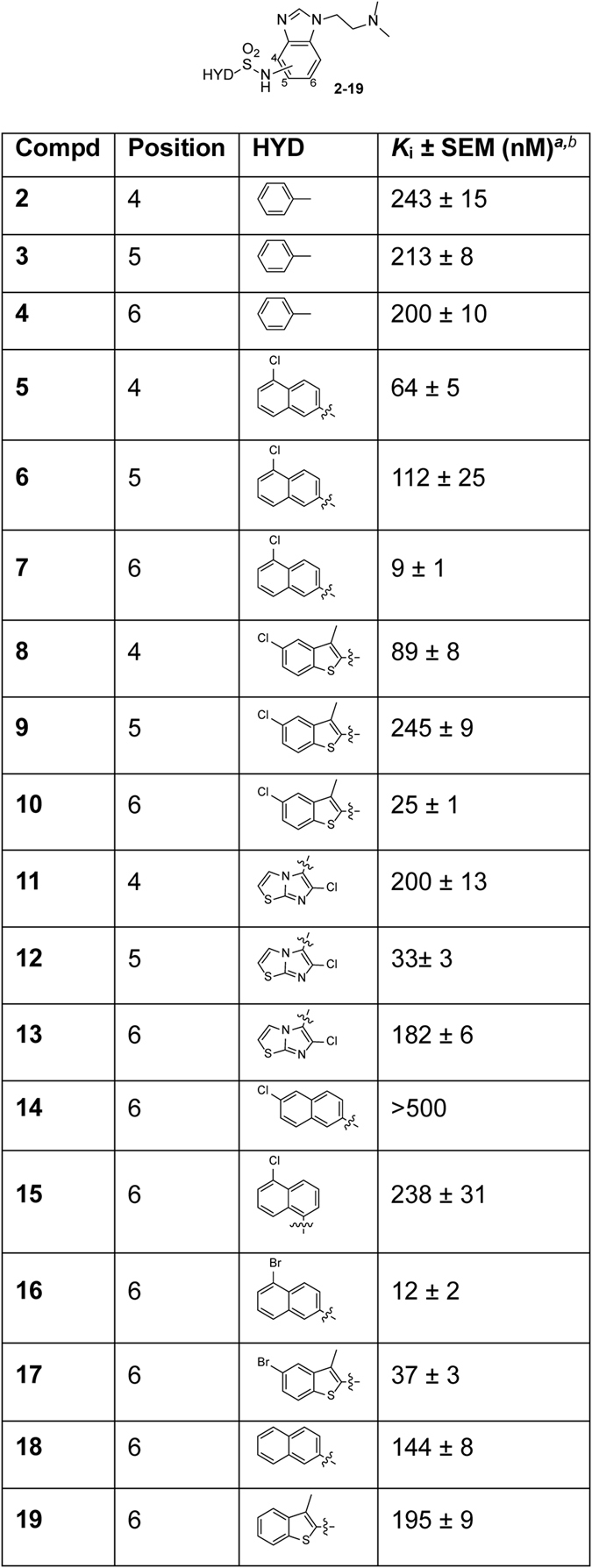
5-HT_6_R affinities of new compounds 2–19. ^*a*^Values are the mean of two to four experiments performed in triplicate. ^*b*^5-HT (*K*_i_ = 71 ± 6 nM) and SB-258585 (*K*_i_ = 4.5 ± 0.2 nM) were used as reference compounds.

In the new compounds (Fig. 1C), a benzene ring was initially maintained as HYD site, while a number of basic moieties were considered as PI element, and different functional groups were explored as HBA feature at the different positions of the central benzimidazole system. Among synthesized compounds (see Supplementary Table S1 for some selected derivatives), only analogues **2–4** containing the NHSO_2_ group as HBA in positions 4–6 of the benzimidazole bound the 5-HT_6_R with moderate affinity (*K*_i_ = 200–243 nM, Supplementary Table S1 and Fig. 2). Hence, we synthesized new related sulfonamides **5–13** bearing a (dimethylamino)ethyl group as PI and open to diverse HYD moieties, based on commonly present systems in 5-HT_6_R antagonists, such as 5-chloro-2-naphthyl, 5-chloro-3-methyl-1-benzothiophen-2-yl, or 6-chloroimidazo[2,1-*b*][1,3]thiazol-5-yl. Additionally, analogues **14–19** were prepared to study the influence of the halogen atom in this class of ligands (Figs 1D and 2).

### Synthesis

**Figure 3:**
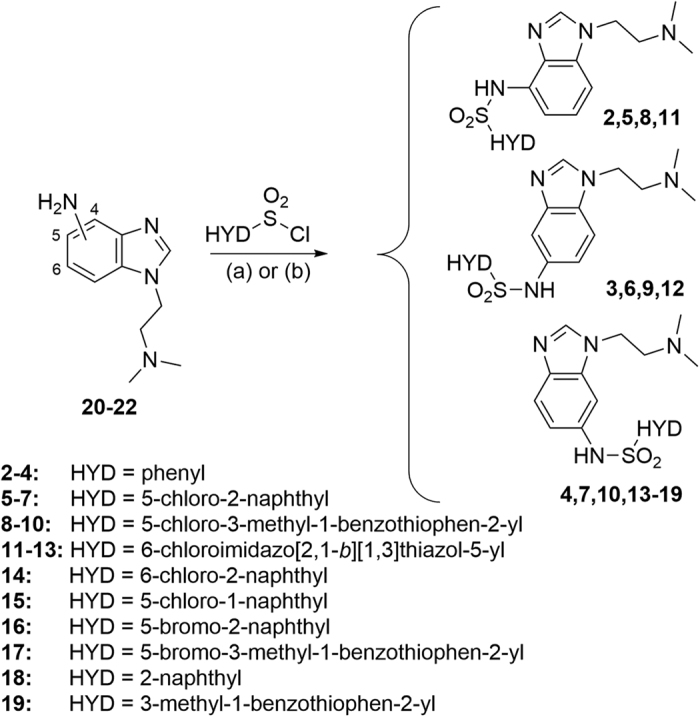
Synthesis of target compounds 2–19. Reagents and conditions: (**a**) pyridine, dichloromethane, rt, 15 h (**2–6**, **8–12**, **14–19**); (**b**) NaHCO_3_, acetonitrile, rt, 22 h (**7**, **13**).

**Figure 4:**
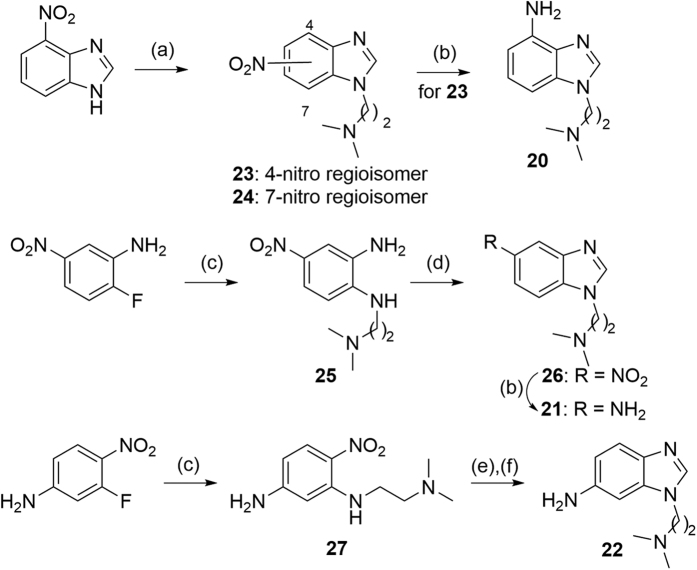
Synthesis of aminobenzimidazoles 20–22. Reagents and conditions: (**a**) i. Cl(CH_2_)_2_N(CH_3_)_2_·HCl, K_2_CO_3_, NaI, DMF, 60 °C, 15 h; ii. column chromatography (SiO_2_, EtOAc/MeOH 98:2), **23**: 33%, **24**: 30%; (**b**) H_2_, 10% Pd(C), MeOH, rt, 15 h, quantitative; (**c**) H_2_N(CH_2_)_2_N(CH_3_)_2_, K_2_CO_3_, DMF, 90 °C, 12 h, 60–78%; (**d**) HCOOH, H_2_O, reflux, 3 h, 92%; (**e**) 10% Pd(C), HCOOH, MeOH, reflux, 20 h, 69%; (**f**) 4 M H_2_SO_4_, THF, 50 °C, 1 h, quantitative.

Target compounds **2–19** were synthesized by reaction of the appropriate aminobenzimidazole **20–22** with the corresponding sulfonyl chloride (Fig. 3). Intermediates **20–22** were obtained according to the synthetic routes detailed in Fig. 4. 4-Nitrobenzimidazole was treated with 2-chloro-*N,N*-dimethylethylamine and the obtained 4- and 7-nitro regioisomers (**23** and **24**, respectively) were separated by column chromatography. Then, pure **23** was reduced to obtain 4-aminobenzimidazole **20**. Nucleophilic substitution of 2-fluoro-5-nitroaniline with *N,N*-dimethylethylenediamine followed by Phillips cyclization of **25** with formic acid and nitro reduction of **26** yielded the corresponding 5-aminobenzimidazole **21**. In the case of 6-aminobenzimidazole **22**, nucleophilic substitution was applied to 3-fluoro-4-nitroaniline, then **27** was cyclized under reductive conditions.

### High-affinity ligands 7 and 10 exhibit antagonist character at the human 5-HT_6_R

Synthesized compounds **2–19** were assessed for *in vitro* affinity at the human 5-HT_6_R by radioligand competitive binding assays, using [^3^H]LSD in membranes of transfected HEK-293 cells (Fig. 2). In general, ligands anchoring the pharmacophore elements (HBA-HYD) at position 6 of the central benzimidazole system exhibited high affinity for the 5-HT_6_R, while positions 4 and 5 were less favorable [**7** (*K*_i_ = 9 nM) vs **5** (*K*_i_ = 64 nM) and **6** (*K*_i_ = 112 nM), **10** (*K*_i_ = 25 nM) vs **8** (*K*_i_ = 89 nM) and **9** (*K*_i_ = 245 nM)]. 6-Benzimidazole derivatives bearing a halogen atom at position 5 of the HYD moiety showed the highest affinity values [**7** (*K*_i_ = 9 nM), **10** (*K*_i_ = 25 nM), **16** (*K*_i_ = 12 nM), and **17** (*K*_i_ = 37 nM)], whereas a drop of affinity was observed for analogues **14** and **15** containing a different substitution pattern in the chloronaphthyl moiety [**14** (*K*_i_ > 500 nM) and **15** (*K*_i_ = 238 nM) vs **7** (*K*_i_ = 9 nM)]. Notably, removal of the halogen atom in HYD moieties was detrimental for 5-HT_6_R affinity in **18** and **19** [**18** (*K*_i_ = 144 nM) vs **7** (*K*_i_ = 9 nM) and **16** (*K*_i_ = 12 nM); **19** (*K*_i_ = 195 nM) vs **10** (*K*_i_ = 25 nM) and **17** (*K*_i_ = 37 nM)].

**Figure 5:**
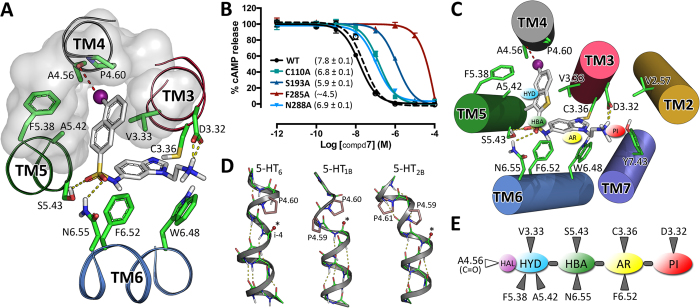
Homology models and site-directed mutagenesis define the binding mode to the 5-HT_6_R. (**A**,**C**) Detailed view of the binding modes of compounds **7** (panel A) and **10** (panel C) in the energy-minimized computational models of the 5-HT_6_R. The amino acids of the receptor (labeled according to Ballesteros GPCR nomenclature) are shown in green and compounds **7** and **10** in white sticks. The pharmacophore elements PI, AR, HBA, and HYD are labeled in panel C according to Fig. 1. Chlorine atoms (purple spheres) of compounds **7** and **10** halogen bond the carbonyl group at position 4.56 (i-4 relative to Pro 4.60, red dashed lines). (**B**) The antagonist effect (pIC_50_ values are shown within the parenthesis) of compound **7** is represented as the percentage of inhibition of 5-HT-induced stimulation of cAMP taken as 100%, in WT and mutant receptors. SB-258585 (pIC_50_ = 7.6 ± 0.1, dashed line) was assayed in WT as a reference compound. Values are represented as mean ± SEM of 3–5 independent experiments each performed in triplicate. (**D**) Main chain hydrogen bond network on the extracellular side of TM 4 in the computational model of the 5-HT_6_R (left) and crystal structures of 5-HT_1B_R (PDB ID 4IAR, middle) and 5-HT_2B_R (PDB ID 4IB4, right). Exposed carbonyl oxygens (small spheres marked with an asterisk) in TM 4 are free to interact with halogen atoms. (**E**) Extension of our previously reported pharmacophore model for 5-HT_6_R antagonists, and the predicted interacting amino acids in the TMs of the 5-HT_6_R.

High-affinity ligands **7** and **10** were assessed for functional activity at the receptor. The effect on adenylate cyclase (AC) activity was evaluated in HEK-293 cells expressing the human 5-HT_6_R. The ligands did not induce a substantial increase in cAMP levels, while a complete disappearance of the 5-HT induced response (≥95%) was attained and a dose-dependent decrease of cAMP concentration was observed for both ligands **7** and **10** (pIC_50_ = 7.8 and 7.3, respectively), as well as for SB-258585 used as reference compound (Fig. 5B). These data indicate that compounds **7** and **10** act as antagonists at the human 5-HT_6_R in the AC assay.

### Homology models and site-directed mutagenesis define the binding mode to the 5-HT_6_R

In order to rationalize the experimental affinity data we developed computational models of the ligand-receptor complexes. To achieve this task, a three-dimensional homology model of the human 5-HT_6_R was constructed from the crystal structure of the closely related 5-HT_2B_ receptor (see Supplementary Figure S3 for sequence alignment), which exhibits conformational characteristics of the inactive state^11^. In our ligand-receptor models (Fig. 5A and C), D106^3.32^ (superscript refers to the Ballesteros–Weinstein nomenclature system) anchors the PI (dimethylamino)ethyl group, the central benzimidazole (AR) interacts with C110^3.36^ and F285^6.52^, the sulfonamide group (HBA) hydrogen bonds N288^6.55^ and S193^5.43^, and the terminal HYD system expands into a hydrophobic cavity between transmembrane domains (TMs) 3, 4, and 5 (surface in Fig. 5A). As shown in Fig. 5A and C, the binding conformation of compounds **7** and **10** is further stabilized by a halogen bond interaction between the chlorine substituent in HYD and the A157^4.56^ backbone carbonyl group (at position i-4 from P^4.60^, see below). Chlorine, as well as bromine and iodine, has a small positively-charged surface (σ-hole) on its hind side along the C−Cl bond axis that permits a Lewis acid-base interaction, in which halogen acts as Lewis acid^12^. These halogen bonds are preferentially formed between the halogen atom of the ligand and the carbonyl oxygen of the protein backbone, which is the most abundant Lewis base in proteins^13^. In this regard, serotonin receptors, as well as other GPCRs, contain a break on the main chain hydrogen bond network in TM 4 caused by proline residues PP^4.60^ (in 50% of the human sequences), PxP^4.61^ (25%) or P^4.60^ (25%). The break is produced to avoid a steric clash between the pyrrolidine ring of Pro (at position i) and the residue in the preceding turn of the helix^14^, leading to an exposed carbonyl oxygen at position i-4 able to interact with the halogen atom. Figure 5D shows P^4.60^ in the computational model of the 5-HT_6_R as well as PP and PxP motifs in the crystal structures of 5-HT_1B_ and 5-HT_2B_ receptors, respectively^10^,^11^. These models explain that in 5-chloro-2-naphthyl and 5-chloro-3-methyl-1-benzothiophen-2-yl derivatives the highest affinity is achieved when the HYD moiety is anchored at the 6-position of the benzimidazole central core [*K*_i_(**7**) = 9 nM vs *K*_i_(**5**) = 64 nM and *K*_i_(**6**) = 112 nM; *K*_i_(**10**) = 25 nM vs *K*_i_(**8**) = 89 nM and *K*_i_(**9**) = 245 nM, respectively] (Fig. 2). The maintenance of nanomolar affinity in bromo-HYD analogues [**16** (*K*_i_ = 12 nM) vs **7** (*K*_i_ = 9 nM) and **17** (*K*_i_ = 37 nM) vs **10** (*K*_i_ = 25 nM)] suggests a subtle equilibrium between volume and σ-hole magnitude. Also, the drop of affinity in the case of non-halogenated derivatives [**18** (*K*_i_ = 144 nM) vs **7** (*K*_i_ = 9 nM) and **19** (*K*_i_ = 195 nM) vs **10** (*K*_i_ = 25 nM)] further supports the importance of a halogen bond interaction in the HYD moiety to stabilize the binding in this class of 5-HT_6_R ligands. Unbiased 1 μs molecular dynamics (MD) simulation was used to study the proposed binding interactions of the high-affinity compound **7** and its non-halogenated analogue, compound **18** with the 5-HT_6_R. Analysis of the MD trajectories suggested that the predicted complex between compound **7** and the 5-HT_6_R is highly stable, with average ligand root-mean-square deviations (RMSDs) = 1.5 Å relative to the initial docking pose. In contrast, compound **18** is less stable (RMSD = 2.2 Å), with larger fluctuations in the HYD group due to the absence of the halogen bond stabilizing effect (see Supplementary Figure S4). The evolution of the halogen bond distance and the σ-hole angle during the MD simulation are reported in Supplementary Figure S5. The average computed values of 3.1 Å and 170°, respectively, are in agreement with bibliographic data^12^.

Experimental validation of the halogen bond interaction with the backbone carbonyl of A157^4.56^ is not straightforward because its mutation to a different amino acid (except Pro) would not modify the chemical composition of the backbone. We, thus, took an indirect approach in which we validated the binding mode of compound **7** by mutating all amino acids predicted to form the binding site in the computational model. Ala substitution of the C110^3.36^ (pIC_50_ = 6.8), S193^5.43^ (5.9), F285^6.52^ (~4.5), and N288^6.55^ (6.9) sites decreases the antagonist activity of compound **7**, relative to wild type (pIC_50_ = 7.8) (Fig. 5B). This agreement between site-directed mutagenesis experiments and computational models points to TMs 3–5 (surface in Fig. 5A) as the cavity to accommodate the terminal HYD moiety of the ligand, and supports the backbone carbonyl group of A157^4.56^, within this cavity, as the putative binding partner for the halogen atom. Altogether, these results allowed us to expand our previously reported pharmacophore model for 5-HT_6_R antagonists^8^. Thus, in addition to the four key structural elements —PI, AR, HBA, and HYD—, we propose the importance of a halogen atom attached to HYD to form a halogen bond with the free carbonyl group at position 4.56 (Fig. 5E).

### New 5-HT_6_R antagonist 7 exhibits procognitive activity

The high-affinity antagonist **7** was selected for the assessment of *in vitro* pharmacokinetic properties. Metabolic stability using rat and human liver microsomes (RLMs and HLMs, respectively) was determined as a measure of first-pass metabolism. Test compound was incubated at a concentration of 1 and 5 μM with RLM and HLM preparations, respectively. The half-life time (t_1/2_) was used to calculate the intrinsic clearance (CL_int_). From the data, microsomal metabolism was found to be more favourable for HLMs (t_1/2_ = 77.5 min and CL_int_ = 8.0 mL/min/kg) than for RLMs, where a high rate of degradation was found (t_1/2_ = 20.7 min and CL_int_ = 67.8 mL/min/kg). An *in vitro* fluorescence-based inhibition assay was conducted with cytochrome P450 2D6 (CYP2D6), one of the most important enzymes involved in drug metabolism, using human recombinant microsomal CYP2D6 enzyme, AMMC {3-[2-(*N,N*-diethyl-*N*-methylamino)ethyl]-7-methoxy-4-methylcoumarin} as substrate, and quinidine as control inhibitor. After 30 min of incubation with compound **7** at a concentration of 10 μM, a remaining activity of 78% of the cytochrome was found.

Compound **7** was also tested for interaction with human serum albumin (HSA), and a binding of 70% was determined at a concentration of 5 μM. Additionally, we evaluated the cell permeability of **7** with the well-validated parallel artificial membrane permeability assay (PAMPA) technique. The compound showed a permeability value (P) of 12 × 10^−6^ cm/s, intermediate between that of propranolol (P = 25 × 10^−6^ cm/s) and metoprolol (P = 9 × 10^−6^ cm/s), both highly permeable drugs used as references. To further assess the potential of lead compound **7** as a drug candidate, hERG inhibition was determined as an indication of possible lethal side effects related with cardiac toxicity. In a hERG whole-cell patch clamp assay **7** showed a low blockade of the K^+^ channel current (IC_50_ > 10 μM, n = 3). Moreover, the selectivity of **7** over the serotonin 5-HT_2B_ receptor (16% displacement of radioligand at 1 μM, Supplementary Table S2) exclude a potential cardiac liability associated to this receptor^15^. Altogether, these *in vitro* studies indicate that the new 5-HT_6_R antagonist **7** identified in this work deserves consideration for further pharmacological characterization.

**Figure 6:**
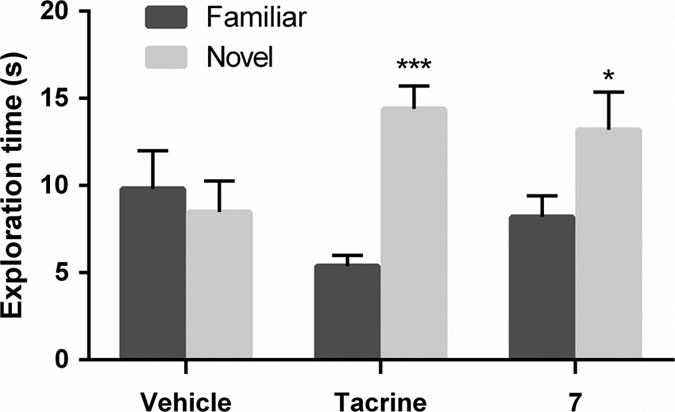
Procognitive activity tested in the novel object recognition task. Effect of compound **7** (1 mg/kg, ip) and cognitive enhancer tacrine (0.5 mg/kg, po) on time-induced memory deficit tested in the NORT in rats. Data are means ± SEM of exploration times; *P < 0.05 and ***P < 0.001 (paired Student’s *t* test) indicate significant differences of time spent exploring the novel vs familiar objects. N = 8–10 animals/group.

Subsequently, the *in vivo* activity of compound **7** was evaluated on the novel object recognition task (NORT) in rats, an animal model widely used to assess memory function. As shown in Fig. 6, statistical analysis of the results revealed a significant effect for treatment with tested compound **7** and tacrine, which was also assayed for comparative purposes. Specifically, 60 min after administration of tacrine (0.5 mg/kg, po) and **7** (1 mg/kg, ip), animals spent more time exploring the novel object than the familiar one in the test phase, whereas no significant difference was observed in the control group treated with vehicle. The enhanced recognition memory during the test phase indicates that compound **7** reversed the time-delay induced memory deficit. These results support the procognitive property of the 5-HT_6_R antagonist newly identified herein, similarly to the behavior observed in NORT for other 5-HT_6_R antagonists such as SB-742457^16^, clinically tested for the treatment of Alzheimer’s disease^5^. The compound was also assessed for binding affinity toward a set of receptors interacting with cognitive enhancer drugs —serotonin 5-HT_1A_, 5-HT_2A_, 5-HT_4e_, and 5-HT_7_, histamine H_3_, muscarinic acetylcholine M_1_, cannabinoid CB_2_, α_2_ adrenergic, α_7_ nicotinic, and NMDA receptors—17,18. In all cases the displacement of the corresponding radioligand by compound **7** (at a concentration of 1 μM) was lower than 23% (see data in Supplementary Table S2), indicating no major selectivity concerns over the tested receptors.

## Discussion

Among the newly synthesized 5-HT_6_R ligands described herein (Figs 2, 3 and 4), compounds **7** and **10** have been pharmacologically characterized as antagonists at the human receptor in the AC assay (see Results section). 5-HT_2B_-based homology models of ligand-receptor complexes (Fig. 5A and C) predicted interactions of the key structural elements —PI ((dimethylamino)ethyl), AR (benzimidazole) and HBA (sulfonamide)— with D106^3.32^, C110^3.36^, F285^6.52^, N288^6.55^, and S193^5.43^ of the 5-HT_6_R binding site in TMs 3–6, and an additional stabilization via a halogen bond interaction between the HYD moiety (5-chloro-2-naphthyl or 5-chloro-3-methyl-1-benzothiophen-2-yl) and the A157^4.56^ backbone carbonyl group within a hydrophobic cavity in TMs 3–5. These models are in agreement with the experimental binding data that revealed the nanomolar affinity exhibited by both chloro- and bromo-HYD analogues **7**, **10**, **16** and **17**, and the drop of affinity in non-halogenated derivatives **18** and **19**. The decrease of the antagonist activity of compound **7** when the C110^3.36^, S193^5.43^, F285^6.52^, and N288^6.55^ were mutated to Ala (Fig. 5B) validated the predicted binding mode to the 5-HT_6_R and supported the backbone carbonyl group of A157^4.56^ as the putative binding partner for the halogen atom. Hence, combined site-directed mutagenesis and computational studies of this class of antagonists in complex with the 5-HT_6_R expand our pharmacophore model; in addition to the four key structural elements —PI, AR, HBA, and HYD—, we propose the importance of a halogen atom attached to the HYD moiety to form a halogen bond with the free carbonyl group at position 4.56 (Fig. 5E).

The *in vitro* ADMET properties —microsomal metabolism, CYP2D6 inhibition, HSA binding, cell permeability, and hERG inhibition— of the 5-HT_6_R antagonist newly identified herein **7** excluded important pharmacokinetic issues and possible lethal side effects related with cardiac toxicity (see Results section). The potential of compound **7** as a drug candidate was explored in the NORT assay in rats that revealed a significant reversal of the time-delay induced memory deficit when administered at 1 mg/kg (ip) (Fig. 6). The observed effect is in agreement with previous studies that have shown that several selective 5-HT_6_R antagonists are endowed with cognitive enhancing capability^19^. In addition, the observed selectivity over other receptors interacting with cognitive enhancer drugs —serotonin 5-HT_1A_, 5-HT_2A_, 5-HT_4e_, and 5-HT_7_, histamine H_3_, muscarinic acetylcholine M_1_, cannabinoid CB_2_, α_2_ adrenergic, α_7_ nicotinic, and NMDA— (Supplementary Table S2) suggests that the procognitive activity of **7** is mediated by the 5-HT_6_R, though an interaction with additional targets can not be discarded.

In conclusion, new benzimidazole-based compounds have been developed as a new class of 5-HT_6_R antagonists. Mutagenesis and homology models have defined the binding mode that includes a halogen bond as an important ligand-receptor interaction. The promising *in vivo* behaviour exhibited by the new selective antagonist **7** further supports that 5-HT_6_R antagonists represent a step toward the identification of new cognitive enhancers for the treatment of AD and other psychiatric and neurological disorders with associated cognitive dysfunction.

## Methods

### Chemistry

Unless stated otherwise, starting materials, reagents and solvents were purchased as high-grade commercial products from Sigma-Aldrich, ABCR, Acros, Fluorochem, or Scharlab, and were used without further purification. Anhydrous tetrahydrofuran (THF) and dichloromethane were dried using a Pure Solv™ Micro 100 Liter solvent purification system. Analytical thin-layer chromatography (TLC) was run on Merck silica gel plates (Kieselgel 60 F-254) with detection by UV light (254 nm), ninhydrin solution, or 10% phosphomolybdic acid solution in ethanol. Flash chromatography was performed on a Varian 971-FP flash purification system using silica gel cartridges (Varian, particle size 50 μm). Melting points (mp, uncorrected) were determined on a Stuart Scientific electrothermal apparatus. Infrared (IR) spectra were measured on a Bruker Tensor 27 instrument equipped with a Specac ATR accessory of 5200-650 cm^−1^ transmission range; frequencies (ν) are expressed in cm^−1^. Nuclear Magnetic Resonance (NMR) spectra were recorded at room temperature on Bruker Avance 500 (^1^H, 500 MHz; ^13^C, 125 MHz) or Bruker Avance 300-AM (^1^H, 300 MHz; ^13^C, 75 MHz) spectrometers at the Universidad Complutense de Madrid (UCM) NMR facilities. Chemical shifts (δ) are expressed in parts per million relative to internal tetramethylsilane; coupling constants (*J*) are in hertz (Hz). The following abbreviations are used to describe peak patterns when appropriate: s (singlet), d (doublet), t (triplet), q (quartet), qt (quintet), m (multiplet), br (broad), and app (apparent). 2D NMR experiments (HMQC and HMBC) of representative compounds were carried out to assign protons and carbons of the new structures and the following abbreviations have been used for the peak assignment: benz (benzimidazole), bzthio (benzothiophene), imthiaz (imidazothiazole), naph (naphthalene), and Ph (phenyl). Elemental analyses (C, H, N or C, H, N, S) were obtained on a LECO CHNS-932 apparatus at the UCM’s analysis services and were within ± 0.4% of the theoretical values, confirming a purity of at least 95% for all tested compounds.

HPLC-MS analysis was performed using an Agilent 1200LC-MSD VL instrument. LC separation was achieved with an Agilent Eclipse XDB-C18 column (5 μm, 4.6 mm × 150 mm) together with a guard column (5 μm, 4.6 mm × 12.5 mm). The gradient elution mobile phases consisted of A (95:5 water:methanol) and B (95:5 methanol:water) with 0.1% formic acid and 0.1% NH_4_OH as the solvent modifiers. MS analysis was performed with an electrospray ionization (ESI) source. The capillary voltage was set to 3.0 kV and the fragmentor voltage was set at 70 eV. The drying gas temperature was 350 °C, the drying gas flow rate was 10 L/min and the nebulizer pressure was 20 psi. MS measurements were made by selected ion monitoring (SIM).

### General procedure for the synthesis of final compounds 2–19

#### General procedure A

To a solution of the corresponding aminobenzimidazole **20–22** in dry dichloromethane (6 mL/mmol), anhydrous pyridine (2 equiv) was added dropwise at room temperature and under an argon atmosphere. Then, the proper arylsulfonyl chloride (1-1.2 equiv) was added and the reaction mixture was stirred for 15 h. The crude was washed with H_2_O and brine, dried (Na_2_SO_4_), filtered, and evaporated. The residue was purified by column chromatography using the appropriate eluent, to provide pure sulfonamides **2**–**6, 8–12**, **14–19**.

#### General procedure B

To a solution of the 6-aminobenzimidazole **22** in dry acetonitrile (5 mL/mmol), NaHCO_3_ (2.8 equiv) was added at room temperature and under an argon atmosphere, and the mixture was stirred for 15 min. Then, the proper arylsulfonyl chloride (1.05 equiv) was added and the reaction was stirred for 22 h. The reaction mixture was filtered and the solvent was evaporated. The residue was purified by column chromatography using the appropriate eluent, to provide pure sulfonamides **7**, **13**.

### 5-Chloro-*N*-{1-[2-(dimethylamino)ethyl]-1*H*-benzimidazol-6-yl}naphthalene-2-sulfonamide (7)

Obtained from the 6-aminobenzimidazole **22** (101 mg, 0.49 mmol) and 5-chloronaphthalene-2-sulfonyl chloride (116 mg, 0.52 mmol) using general procedure B in 34% yield (71 mg). Chromatography: dichloromethane to dichloromethane/ethanol, 7:3; mp 170 °C (decomposes); IR (ATR) ν 3429, 3064, 1625, 1592, 1501, 1466, 1322; ^1^H NMR (300 MHz, DMSO-*d*_*6*_) δ 2.00 (s, 6 H, 2CH_3_), 2.38 (t, *J* = 6.1, 2 H, CH_2_NMe_2_), 4.16 (t, *J* = 6.1, 2 H, CH_2_N), 6.92 (dd, *J* = 8.6, 1.8, 1 H, CH_benz_), 7.22 (d, *J* = 1.8, 1 H, CH_benz_), 7.45 (d, *J* = 8.6, 1 H, CH_benz_), 7.60 (t, *J* = 8.0, 1 H, CH_naph_), 7.85 (d, *J* = 6.9, 1 H, CH_naph_), 7.94 (dd, *J* = 9.0, 1.8, 1 H, CH_naph_), 8.08–8.12 (m, 2 H, CH_benz_, CH_naph_), 8.31 (d, *J* = 9.0, 1 H, CH_naph_), 8.47 (d, *J* = 1.8, 1 H, CH_naph_), 10.37 (br s, 1 H, NH); ^13^C NMR (75 MHz, DMSO-*d*_*6*_) δ 42.1 (CH_2_), 44.9 (2CH_3_), 57.7 (CH_2_), 103.5, 116.8, 119.7, 123.9, 125.4, 128.0, 128.5, 128.9, 129.1 (9CH), 130.6, 131.1, 132.0, 132.9, 133.7, 137.6, 140.8 (7 C), 144.8 (CH); MS (ESI) 429.2 [M(^35^Cl) + H]^+^, 431.2 [M(^37^Cl) + H]^+^. Anal. (C_21_H_21_ClN_4_O_2_S) C, H, N, S.

### 5-HT_6_R radioligand binding assay

Competitive inhibition assays were carried out following previously reported procedure^20^.

### 5-HT_6_R AC assay

Experimental details for cell cultures, transfection, expression vectors, mutagenesis, and cAMP measurements are fully described in the Supporting Information. Briefly, 24 h after transfection, cells were incubated in Dulbecco’s modified Eagle’s serum-free medium for 4 h. Prior to the experiments, cell culture medium was replaced and plates were equilibrated for 1 h with 100 μL of assay medium at room temperature in the dark. The assay medium consisted in Hank’s Balanced Salt Solution (HBSS) with 24 mM HEPES, 3.3 mM Na_2_CO_3_, 1.3 mM CaCl_2_, 1 mM MgSO_4_, 0.1% (w/v) bovine serum albumin and 0.45 mg/mL D-Luciferin (all reagents were purchased from Sigma-Aldrich, UK, with the exception of D-Luciferin, Nanolight^®^ Technology, USA). Bioluminescence was quantified using a CLARIOstar^®^ Multimode Plate Reader (BMG Labtech, Germany) with 1 s integration time/well. Prior to treatment of cells, 5 measurements were made, at 2-min intervals, to determine the basal luminescence levels. To account for differences in expression/cell density, the average of these 5 pre-readings was used to normalize each well’s response. To determine the potency of 5-HT for 5-HT_6_ wild type and mutant receptors, different concentrations of 5-HT or tested compound were added and 20 measurements were taken in kinetic mode every 2 min. For real-time competition experiments, tested compounds were pre-incubated for 20 min prior to 5-HT stimulation (10 × 5-HT EC_50_ for each receptor form to ensure > 90% receptor occupancy, see Supplementary Figure S2). The area under the curve was used to fit the dose-response curves by nonlinear regression using a three parameters logistic equation. Data were analyzed using GraphPad Prism 6.0 h (GraphPad Software, La Jolla, CA).

### *In vitro* pharmacokinetics

RLM and HLM stability, HSA binding, and fluorescence-based CYP2D6 inhibition assays were performed according to previously reported procedures^21^. Inhibition of hERG K^+^ channel current was determined in an automated patch clamp assay using whole CHO-K1 cells at Eurofins Panlab (USA), according to previously published method^22^.

The assessment of the membrane permeability of ligand **7** and reference compounds propranolol and metoprolol was performed in a commercially available 96-well Corning Gentest pre-coated PAMPA plate system (Cultek S.L.U., Spain). Prior to use, the pre-coated PAMPA plate system was warmed to room temperature for 30 min and 300 μL of 200 μM solution of tested compound in 2% DMSO in phosphate buffered saline (PBS, pH 7.4) were added into wells in the receiver (donor) plate. Then 200 μL of PBS were added into wells in the filter (acceptor) plate. The filter plate was placed on the receiver plate by slowly lowering the pre-coated PAMPA plate until it sits on the receiver plate. The assembly was incubated at 25 °C for 5 h, and then buffer samples were collected carefully from each plate. The final concentrations of compound in both donor and acceptor wells were analyzed by HPLC-MS and quantification was estimated by using the peak area integration normalized with an internal standard. Permeability value of the compounds was calculated using the following formula: P (cm/s) = {−ln[1 − C_A_(t)/C_eq_]}/[A*(1/V_D_ + 1/V_A_)*t], where A = filter area (0.3 cm^2^), V_D_ = donor well volume (0.3 mL), V_A_ = acceptor well volume (0.2 mL), t = incubation time (s), C_A_(t) = compound concentration (μM) in acceptor well at time t, C_D_(t) = compound concentration (μM) in donor well at time t, and C_eq_ = [C_D_(t)*V_D_ + C_A_(t)*V_A_]/(V_D_ + V_A_). Assays were performed in duplicate and the compounds were tested in two different plates on different days.

### NORT assay

The novel object recognition task was conducted at Suven Life Sciences (Hyderabad, India) according to previously reported method^19^. All animal care and experiments were carried out in compliance with Institutional Animal Ethics Committee (IAEC) requirements and in-house animal care and usage policy. Every effort was made to reduce the number of animals used and to minimize potential suffering. The experiment was carried out using male Wistar rats (220–280 g) over a period of three days. Briefly, on day 1 (habituation session) the rats were habituated for 20 min to the experimental conditions and then returned to their respective home cages. On day 2 (acquisition session) the rats were allowed to explore two similar objects for 3 min. After this familiarization phase, animals were returned to their home cages. On day 3 (recognition session), 24 h after the familiarization phase, one object presented during the acquisition session was replaced by a new object. The rats were allowed to explore the familiar and the novel objects for 3 min. The times spent by each animal investigating each of the objects were recorded separately in both acquisition and recognition sessions. Exploration times of ≥15 s during the acquisition phase and ≥10 s during the recognition phase were considered for statistical analysis. Compound **7** (1 mg/kg, ip) or tacrine (0.5 mg/kg, po) or vehicle (2 mL/kg, ip) were administered 60 min before each session of the test.

### Computational model of ligand-receptor complexes

MODELLER v9.12^23^ was used to build a homology model of human 5-HT_6_R (Uniprot code P50406) (one hundred molecular models were generated) using the three-dimensional crystal structure of the 5-HT_2B_R (PDB code 4IB4) as template^10^. Sequences were aligned using the highly conserved residues shared within the GPCR family (see Supplementary Figure S3)^24^. Loops regions were optimized through a MD Simulated Annealing protocol. For this purpose, backbone residues of TMs and Helix 8 were constrained and the conformation of loops were optimized in 20 Simulated Annealing cycles of heating up to 700 K and slowly cooling down to 300 K in successive 10 K, 100 ps steps followed by an energy minimization. Compounds **7**, **10** and **18** were docked into the receptor model using the AutoDock Vina tool^25^. All docking solutions were visually inspected and the poses in which the protonated amine forms an ionic interaction with D106^3.32^ and the NHSO_2_ group hydrogen bonds N288^6.55^ were energy minimized. The binding modes of compounds **7** and **18** to the 5-HT_6_R were further studied in explicit membrane MD simulations with the GROMACS v4.5.3 simulation package^26^ (see Supplementary Figures S4–S6). Molecular systems were subjected to 50 ns of equilibration, with positional restraints on the backbone atoms of the receptor. These restraints were released, and 1 μs MD trajectory was produced at constant pressure and temperature, using the particle mesh Ewald method to evaluate electrostatic interactions. The AMBER99SB-ILDN force field was used for the protein, Berger parameters for the lipids, and the general Amber force field (GAFF) and HF/6–31 G*-derived RESP atomic charges for the ligand. This procedure has been previously validated^27^. The chlorine atom of compound **7** was parameterized according to the positive-extra-point approach proposed by Ibrahim^28^. As a result, two point charges separated by 1.9 Å (a negative charge [−0.0725e] centered on the halogen atom to reproduce the electronegative crown, and a positive charge [+0.04e] centered on a dummy atom on the hind side of the C−Cl bond axis in order to reproduce the positively-charged σ-hole) were used to account for the potential effect of halogen bonding in the standard force field^29^.

## Additional Information

**How to cite this article:** González-Vera, J. A. *et al*. A new serotonin 5-HT_6_ receptor antagonist with procognitive activity - Importance of a halogen bond interaction to stabilize the binding. *Sci. Rep.* **7**, 41293; doi: 10.1038/srep41293 (2017).

**Publisher's note:** Springer Nature remains neutral with regard to jurisdictional claims in published maps and institutional affiliations.

## Supplementary Material

Supporting Information
