# Supplementary material for: A new serotonin 5-HT6 receptor antagonist with procognitive activity – Importance of a halogen bond interaction to stabilize the binding
Source: Sci Rep. 2017 Jan 24;7:41293. doi: 10.1038/srep41293 (PMC5259792; doi:10.1038/srep41293)
Supplement: Supporting Information [file srep41293-s1.doc]

**A new serotonin 5-HT_6_ receptor antagonist with procognitive activity – Im-portance of a halogen bond interaction to stabilize the binding**

**Juan A. González-Vera,^1,‡,⊥^ Rocío A. Medina,^1,⊥^ Mar Martín-Fontecha,^1^ Angel Gonzalez,^2^ Tania de la Fuente,^1,‖^ Henar Vázquez-Villa,^1^ Javier García-Cárceles,^1^ Joaquín Botta,^3^ Peter J. McCormick,^3^ Bellinda Benhamú,^1^ Leonardo Pardo,^2,*^ and María L. López-Rodríguez^1,*^**

^1^Departamento de Química Orgánica I, Facultad de Ciencias Químicas, Universidad Complutense de Madrid, E-28040 Madrid, Spain, ^2^Laboratori de Medicina Computacional, Unitat de Bioestadística, Facultat de Medicina, Universitat Autònoma de Barcelona, E-08193 Bellaterra, Spain, ^3^School of Pharmacy, University of East Anglia, NR4 7TJ Norwich, UK.

**Supporting Information**

**Contents**

1. Synthesis, Affinity and Characterization Data of Compounds **S1-S11** Page S2

2. Synthesis and Characterization Data of Compounds **20-22, 30** Page S6

3. Characterization Data of Final Compounds **2-6**, **8-19** Page S8

4. Elemental Analysis Data Page S13

5. Affinity Data of Compound **7** toward a Panel of Receptors Page S14

6. Mutagenesis Assays Page S14

7. Sequence Alignment and Binding Pocket Analysis of Serotonin Receptors Page S16

8. MD Simulations of Compound **7** and **18** in Complex with the 5-HT_6_R Page S17

9. MD Trajectory Analysis of Compound **7** in Complex with the 5-HT_6_R Page S18

10. Molecular Systems used in Explicit Membrane MD Simulations Page S19

**1. Synthesis, Affinity and Characterization Data of Compounds S1-S11**

| **Compound** | **X** | **Position** | **n** | **R^1^** | **R^2^** | *K*_i_ (5-HT_6_), nM |
| --- | --- | --- | --- | --- | --- | --- |
| **S1** | CONH | 4 | 3 | Me | Me | > 500 |
| **S2** | CONH | 5 | 3 | Me | Me | > 500 |
| **S3** | CONH | 5 | 2 | *i*-Pr | *i*-Pr | > 500 |
| **S4** | CONH | 6 | 2 | *i*-Pr | *i*-Pr | > 500 |
| **S5** | CONH | 5 | 2 | -(CH_2_)_5_- | | > 500 |
| **S6** | CONH | 6 | 2 | -(CH_2_)_5_- | | > 500 |
| **S7** | NHCO | 4 | 2 | Me | Me | > 500 |
| **S8** | NHCO | 5 | 2 | Me | Me | > 500 |
| **S9** | NHCO | 6 | 2 | Me | Me | > 500 |
| **S10** | NHCO | 7 | 2 | Me | Me | > 500 |
| **2** | NHSO_2_ | 4 | 2 | Me | Me | 243± 15 |
| **3** | NHSO_2_ | 5 | 2 | Me | Me | 213 ± 8 |
| **4** | NHSO_2_ | 6 | 2 | Me | Me | 200± 10 |
| **S11** | NHSO_2_ | 7 | 2 | Me | Me | > 500 |

**Supplementary Table S1.** Structure and 5-HT_6_R affinities of analogues **S1-S11, 2-4**, selected from compounds represented in Figure 1

**Supplementary Figure S1.** Synthesis of Compounds **S1-S11**. Reagents and conditions: (a) i) SOCl_2_, toluene, reflux, 5 h; ii) PhNH_2_, THF, rt, 15 h, 71-75%; (b) K_2_CO_3_, NaI, DMF, 60 ºC, 15 h, 21-49%; (c) PhCOOH, EDC, HOBt, DMF, rt, 3 h, 80-89%; (d) PhSO_2_Cl, pyridine, dichloromethane, rt, 15 h, 73%.

**General Procedure for the Synthesis of Carboxamides 28 and 29.** To a suspension of the corresponding benzimidazolecarboxylic acid (1 equiv) in dry toluene (3.2 mL/mmol), SOCl_2_ (11 equiv) was added dropwise and under an argon atmosphere. The reaction mixture was stirred at reflux for 5 h. Once at room temperature, the excess of SOCl_2_ and the solvent were evaporated under reduced pressure and the resulting acid chloride was dissolved in anhydrous THF (3.2 mL/mmol). Then, aniline (1 equiv) was added dropwise at 0 ºC and the reaction mixture was stirred at reflux for 15 h. The solvent was evaporated under reduced pressure and the residue was treated with a saturated solution of NaHCO_3_ and extracted with EtOAc (3 x 25 mL). The organic layers were washed with water, dried (Na_2_SO_4_), filtered and evaporated. The residue was purified by column chromatography to afford amides **28** or **29**.

***N*-Phenyl-1*H*-benzimidazole-4-carboxamide (28).** Obtained from 1*H*-benzimidazole-4-carboxylic acid (614 mg, 3.79 mmol), SOCl_2_ (2.5 mL, 34.4 mmol), and aniline (0.28 mL, 3.10 mmol) in 71% yield (522 mg). Chromatography: dichloromethane to dichloromethane/EtOH 9:1; mp 99-101 ºC; IR (ATR) ν 3140, 3046, 1657, 1607, 1556, 1490; ^1^H NMR (300 MHz, DMSO-*d*_6_) δ 7.15 (t, *J* = 7.5, 1H, CH_Ph_), 7.40-7.44 (m, 3H, CH_benz_, 2CH_Ph_), 7.81 (d, *J* = 7.7, 2H, 2CH_Ph_), 7.85 (d, *J* = 8.0, 1H, CH_benz_), 8.00 (d, *J* = 7.5, 1H, CH_benz_), 8.61 (s, 1H, CH_benz_), 12.20 (br s, 1H, NH), 13.15 (br s, 1H, NH); ^13^C NMR (75 MHz, DMSO-*d*_6_) δ 117.0 (CH), 120.4 (2CH), 123.0 (C), 123.4, 123.6, 124.6 (3CH), 129.9 (2CH), 134.4, 139.8, 141.4 (3C), 144.0 (CH), 163.9 (C).

***N*-Phenyl-1*H*-benzimidazole-5-carboxamide (29).** Obtained from 1*H*-benzimidazole-5-carboxylic acid (1.00 g, 6.17 mmol), SOCl_2_ (5 mL, 68.5 mmol), and aniline (0.56 mL, 6.17 mmol) in 75% yield (1.10 g). Chromatography: dichloromethane/EtOH from 9.5:0.5 to 8:2; mp 120-123 ºC; IR (ATR) ν 3441, 2923, 1637, 1599, 1534, 1501, 1463; ^1^H NMR (300 MHz, CD_3_OD) δ 7.12 (t, *J* = 7.4, 1H, CH_Ph_), 7.37 (t, *J* = 7.9, 2H, 2CH_Ph_), 7.79 (d, *J* = 7.6, 2H, 2CH_Ph_), 7.94 (d, *J* = 8.6, 1H, CH_benz_), 8.14 (dd, *J* = 8.7, 1.5, 1H, CH_benz_), 8.45 (d, *J* = 0.9, 1H, CH_benz_), 9.59 (s, 1H, CH_benz_), 10.57 (br s, 1H, NH), 12.10 (br s, 1H, NH); ^13^C NMR (75 MHz, CD_3_OD) δ 115.6 (CH), 122.4 (2CH), 124.2, 126.0, 127.3 (3CH), 129.9 (2CH), 131.9, 134.3, 135.3, 139.7 (4C), 143.1 (CH), 167.4 (C).

**General Procedure for the Synthesis of Amides S1-S6.** To a suspension of benzimidazole **28** or **29** (1 equiv) and the corresponding chloro-*N,N*-dialkylalkylamine hydrochloride (1.5 equiv) in anhydrous DMF (5 mL/mmol), K_2_CO_3_ (5 equiv) and NaI (1.5 equiv) were added under an argon atmosphere. The reaction mixture was stirred at 60 ºC for 15 h. Once at room temperature, the mixture was filtered, washed with EtOAc and the solvents were evaporated under reduced pressure. The residue was treated with water (50 mL) and extracted with EtOAc (3 x 40 mL). The organic layers were washed with brine, dried (Na_2_SO_4_), filtered and evaporated. The residue was purified by flash chromatography to afford the corresponding *N*-alkylated benzimidazoles **S1-S6**.

**1-[3-(Dimethylamino)propyl]-*N*-phenyl-1*H*-benzimidazole-4-carboxamide (S1).** Obtained from benzimidazole **28** (80 mg, 0.34 mmol) and 3-chloro-*N,N*-dimethyl-1-propanamine hydrochloride (80 mg, 0.51 mmol) in 49% yield (53 mg). Chromatography: hexane/EtOAc from 8:2 to 2:8; mp 104-106 ºC; IR (ATR) ν 3257, 3088, 1676, 1600, 1557, 1495, 1461; ^1^H NMR (300 MHz, CDCl_3_) δ 2.02 (qt, *J* = 6.6, 2H, CH_2_), 2.21 (t, *J* = 6.6, 2H, CH_2_NMe_2_), 2.23 (s, 6H, 2CH_3_), 4.34 (t, *J* = 6.6, 2H, CH_2_N), 7.12 (tt, *J* = 7.3, 1.1, 1H, CH_Ph_), 7.34-7.47 (m, 3H, CH_benz_, 2CH_Ph_), 7.61 (dd, *J* = 8.1, 1.1, 1H, CH_benz_), 7.89 (dd, *J* = 8.6, 1.2, 2H, 2CH_Ph_), 8.04 (s, 1H, CH_benz_), 8.24 (dd, *J* = 7.5, 1.1, 1H, CH_benz_); ^13^C NMR (75 MHz, CDCl_3_) δ 27.7, 43.1 (2CH_2_), 45.6 (2CH_3_), 55.8 (CH_2_), 114.0 (CH), 120.7 (2CH), 123.4, 124.2 (2CH), 124.3 (C), 124.6 (CH), 129.3 (2CH), 134.4, 139.6, 141.6 (3C), 143.5 (CH), 163.9 (C); MS (ESI) 323.3 [M+H]^+^; Anal. (C_19_H_22_N_4_O) C, H, N.

**1-[3-(Dimethylamino)propyl]-*N*-phenyl-1*H*-benzimidazole-5-carboxamide (S2).** Obtained from benzimidazole **29** (68 mg, 0.29 mmol) and 3-chloro-*N,N*-dimethyl-1-propanamine hydrochloride (70 mg, 0.44 mmol) in 46% yield (43 mg). Chromatography: dichloromethane/EtOH from 9:1 to 7:3; mp 108-110 ºC; IR (ATR) ν 3210, 1664, 1599, 1538, 1500, 1462; ^1^H NMR (300 MHz, CD_3_OD) δ 2.11 (qt, *J* = 7.0, 2H, CH_2_), 2.23 (s, 6H, 2CH_3_), 2.34 (t, *J* = 7.2, 2H, CH_2_NMe_2_), 4.42 (t, *J* = 6.9, 2H, CH_2_N), 7.16 (t, *J* = 7.3, 1, 1H, CH_Ph_), 7.38 (t, *J* = 7.5, 2H, 2CH_Ph_), 7.70-7.77 (m, 3H, CH_benz_, 2CH_Ph_), 7.97 (d, *J* = 8.4, 1H, CH_benz_), 8.33 (s, 1H, CH_benz_), 8.35 (s, 1H, CH_benz_); ^13^C NMR (75 MHz, CD_3_OD) δ 27.9, 43.2 (2CH_2_), 45.7 (2CH_3_), 54.3 (CH_2_), 110.7, 120.1 (2CH), 121.3 (2CH), 123.5, 124.3 (2CH), 129.3 (2CH), 130.1, 135.9, 139.6, 143.1 (4C), 146.3 (CH), 166.8 (C); MS (ESI) 323.2 [M+H]^+^; Anal. (C_19_H_22_N_4_O) C, H, N.

**1-[2-(Diisopropylamino)ethyl]-*N*-phenyl-1*H*-benzimidazole-5- and 6-carboxamide (S3 and S4).** Obtained 166 mg (22%) of 5-carboxamide **S3** and 163 mg (21%) of 6-carboxamide **S4** from benzimidazole **29** (500 mg, 2.10 mmol) and (2-chloroethyl)diisopropylamine hydrochloride (652 mg, 3.16 mmol). Chromatography: dichloromethane/EtOH from 99:1 to 9:1.

**S3**: mp 147-148 ºC; IR (ATR) ν 3388, 1655, 1598, 1538, 1500, 1440; ^1^H NMR (300 MHz, CDCl_3_) δ 0.85 (d, *J* = 6.5, 12H, 4CH_3_), 2.76 (t, *J* = 6.0, 2H, CH_2_N*i*-Pr_2_), 2.94 (sept, *J* = 6.5, 2H, 2CH(CH_3_)_2_), 4.09 (t, *J* = 6.0, 2H, CH_2_N), 7.15 (t, *J* = 7.3, 1H, CH_Ph_), 7.38 (app t, *J* = 7.9, 2H, 2CH_Ph_), 7.44 (d, *J* = 8.5, 1H, CH_benz_), 7.78 (d, *J* = 8.1, 2H, 2CH_Ph_), 7.92 (s, 1H, CH_benz_), 7.98 (dd, *J* = 8.5, 1.5, 1H, CH_benz_), 8.12 (br s, 1H, NH), 8.44 (s, 1H, CH_benz_); ^13^C NMR (75 MHz, CDCl_3_) δ 21.1 (4CH_3_), 44.9, 46.5 (2CH_2_), 48.9 (2CH), 110.2, 119.6 (2CH), 120.8 (2CH), 123.4, 124.6 (2CH), 129.5 (2CH), 129.7, 136.5, 139.2, 143.4 (4C), 146.0 (CH), 167.0 (C); MS (ESI) 387.0 [M+Na]^+^; Anal. (C_22_H_28_N_4_O) C, H, N.

**S4**: mp 145-146 ºC; IR (ATR) ν 3444, 1650, 1601, 1540, 1500, 1441; ^1^H NMR (300 MHz, CDCl_3_) δ 0.86 (d, *J* = 6.5, 12H, 4CH_3_), 2.79 (t, *J* = 6.0, 2H, CH_2_N*i*-Pr_2_), 2.97 (sept, *J* = 6.5, 2H, 2CH(CH_3_)_2_), 4.12 (t, *J* = 6.0, 2H, CH_2_N), 7.16 (t, *J* = 7.3, 1H, CH_Ph_), 7.38 (app t, *J* = 7.9, 2H, 2CH_Ph_), 7.69-7.72 (m, 3H, CH_benz_, 2CH_Ph_), 7.80 (d, *J* = 8.4, 1H, CH_benz_), 8.02 (s, 1H, CH_benz_), 8.13 (s, 1H, CH_benz_), 8.30 (br s, 1H, NH); ^13^C NMR (75 MHz, CDCl_3_) δ 20.6 (4CH_3_), 44.5, 45.8 (2CH_2_), 48.4 (2CH), 110.3, 119.8, 120.0 (3CH), 120.3 (2CH), 124.3 (CH), 129.0 (2CH), 129.3, 133.9, 138.2, 146.2 (4C), 146.3 (CH), 166.0 (C); MS (ESI) 387.0 [M+Na]^+^; Anal. (C_22_H_28_N_4_O) C, H, N.

***N*-Phenyl-1-(2-piperidin-1-ylethyl)-1*H*-benzimidazole-5- and 6-carboxamide (S5 and S6).** Obtained 188 mg (40%) of 5-carboxamide **S5** and 190 mg (40%) of 6-carboxamide **S6** from benzimidazole **29** (320 mg, 1.35 mmol) and 1-(2-chloroethyl)piperidine hydrochloride (372 mg, 2.02 mmol). Chromatography: dichloromethane/EtOH from 99:1 to 9:1.

**S5**: mp 147-148 ºC; IR (ATR) ν 3277, 1654, 1618, 1598, 1538, 1500, 1440; ^1^H NMR (300 MHz, CDCl_3_) δ 1.38-1.58 (m, 6H, (CH_2_)_3_), 2.40-2.43 (m, 4H, CH_2_NCH_2_), 2.70 (t, *J* = 6.2, 2H, CH_2_N_piperidine_), 4.24 (t, *J* = 6.3, 2H, CH_2_N), 7.15 (t, *J* = 7.4, 1H, CH_Ph_), 7.39 (app t, *J* = 7.9, 2H, 2CH_Ph_), 7.47 (d, *J* = 8.5, 1H, CH_benz_), 7.75 (d, *J* = 8.4, 2H, 2CH_Ph_), 7.95 (dd, *J* = 8.4, 1.6, 1H, CH_benz_), 8.08 (s, 1H, CH_benz_), 8.41 (s, 1H, CH_benz_), 8.78 (br s, 1H, NH); ^13^C NMR (75 MHz, CDCl_3_) δ 24.3 (CH_2_), 26.1 (2CH_2_), 43.0 (CH_2_), 54.9 (2CH_2_), 58.0 (CH_2_), 110.0, 119.3 (2CH), 120.6 (2CH), 123.2, 124.3 (2CH), 129.1 (2CH), 129.5, 136.4, 138.8, 143.0 (4C), 145.4 (CH), 166.6 (C); MS (ESI) 371.0 [M+Na]^+^; Anal. (C_21_H_24_N_4_O) C, H, N.

**S6**: mp 144-145 ºC; IR (ATR) ν 3442, 1647, 1599, 1537, 1499, 1440; ^1^H NMR (300 MHz, CDCl_3_) δ 1.44-1.62 (m, 6H, (CH_2_)_3_), 2.42-2.46 (m, 4H, CH_2_NCH_2_), 2.73 (t, *J* = 6.3, 2H, CH_2_N_piperidine_), 4.30 (t, *J* = 6.3, 2H, CH_2_N), 7.17 (td, *J* = 7.4, 1.1, 1H, CH_Ph_), 7.39 (app t, *J* = 7.9, 2H, 2CH_Ph_), 7.69-7.73 (m, 3H, CH_benz_, 2CH_Ph_), 7.83 (d, *J* = 8.4, 1H, CH_benz_), 8.02 (s, 1H, CH_benz_), 8.12-8.16 (m, 2H, CH_benz_, NH); ^13^C NMR (75 MHz, CDCl_3_) δ 24.3 (CH_2_), 26.1 (2CH_2_), 43.0 (CH_2_), 54.9 (2CH_2_), 58.0 (CH_2_), 110.5, 119.7, 120.0 (3CH), 120.6 (2CH), 124.6 (CH), 129.1 (2CH), 129.7, 134.0, 138.5, 146.1 (4C), 146.2 (CH), 166.4 (C); MS (ESI) 371.0 [M+Na]^+^; Anal. (C_21_H_24_N_4_O) C, H, N.

**General Procedure for the Synthesis of Amides S7-S10.** To a solution of benzoic acid (1 equiv) in anhydrous DMF (4 mL/mmol), 1-hydroxybenzotriazole (1.2 equiv) and 1-ethyl-3-(3-dimethylaminopropyl)carbodiimide hydrochloride (1.2 equiv) were added and the mixture was stirred at room temperature for 1 h under an argon atmosphere. Then, a solution of the corresponding aminobenzimidazole **20-22** or **30** (1.3 equiv) in anhydrous DMF (2 mL/mmol) was added and the reaction mixture was stirred for 3 h. The solvent was evaporated under reduced pressure and the residue was dissolved in EtOAc and washed with water and brine. The organic layer was dried (Na_2_SO_4_), filtered and evaporated. The residue was purified by flash chromatography to afford the corresponding amides **S7-S10**.

***N*-{1-[2-(Dimethylamino)ethyl]-1*H*-benzimidazol-4-yl}benzamide (S7).** Obtained from 4-aminobenzimidazole **20** (194 mg, 0.95 mmol) in 89% yield (200 mg). Chromatography: dichloromethane/EtOH from 9.5:0.5 to 9:1; mp 109-112 ºC; IR (ATR) ν 3406, 3061, 1671, 1623, 1594, 1532, 1491, 1458; ^1^H NMR (300 MHz, CDCl_3_) δ 2.32 (s, 6H, 2CH_3_), 2.76 (t, *J* = 6.6, 2H, CH_2_NMe_2_), 4.28 (t, *J* = 6.6, 2H, CH_2_N), 7.18 (d, *J* = 8.1, 1H, CH_benz_), 7.36 (t, *J* = 8.0, 1H, CH_benz_), 7.38-7.57 (m, 3H, 3CH_Ph_), 7.96 (s, 1H, CH_benz_), 8.00 (d, *J* = 7.9, 2H, 2CH_Ph_), 8.43 (d, *J* = 7.8, 1H, CH_benz_), 9.18 (br s, 1H, NH); ^13^C NMR (75 MHz, CDCl_3_) δ 43.5 (CH_2_), 45.6 (2CH_3_), 58.5 (CH_2_), 105.0, 111.2, 124.0 (3CH), 127.3 (2CH), 128.8 (2CH), 130.2 (C), 131.9 (CH), 133.8, 134.3, 134.9 (3C), 142.0 (CH), 165.6 (C); MS (ESI) 309.8 [M+H]^+^; Anal. (C_18_H_20_N_4_O) C, H, N.

***N*-{1-[2-(Dimethylamino)ethyl]-1*H*-benzimidazol-5-yl}benzamide (S8).** Obtained from 5-aminobenzimidazole **21** (82 mg, 0.40 mmol) in 83% yield (103 mg). Chromatography: dichloromethane/EtOH from 9.5:0.5 to 9:1; mp 113-115 ºC; IR (ATR) ν 3270, 1655, 1596, 1545, 1492; ^1^H NMR (300 MHz, CDCl_3_) δ 2.31 (s, 6H, 2CH_3_), 2.74 (t, *J* = 6.6, 2H, CH_2_NMe_2_), 4.26 (t, *J* = 6.6, 2H, CH_2_N), 7.42 (d, *J* = 8.5, 1H, CH_benz_), 7.49-7.60 (m, 3H, 3CH_Ph_), 7.75 (dd, *J* = 8.5, 2.0, 1H, CH_benz_), 7.90-7.96 (m, 3H, CH_benz_, 2CH_Ph_), 8.01 (s, 1H, CH_benz_), 8.21 (br s, 1H, NH); ^13^C NMR (75 MHz, CDCl_3_) δ 43.4 (CH_2_), 45.6 (2CH_3_), 58.6 (CH_2_), 109.6, 112.4, 117.5 (3CH), 127.1 (2CH), 128.8 (2CH), 131.4 (C), 131.8 (CH), 132.8, 135.2, 144.0 (3C), 144.2 (CH), 165.9 (C); MS (ESI) 309.8 [M+H]^+^; Anal. (C_18_H_20_N_4_O) C, H, N.

***N*-{1-[2-(Dimethylamino)ethyl]-1*H*-benzimidazol-6-yl}benzamide (S9).** Obtained from 6-aminobenzimidazole **22** (270 mg, 1.32 mmol) in 80% yield (326 mg). Chromatography: dichloromethane/EtOH from 9.5:0.5 to 9:1; mp 115-117 ºC; IR (ATR) ν 3268, 1654, 1601, 1543, 1495; ^1^H NMR (300 MHz, CDCl_3_) δ 2.35 (s, 6H, 2CH_3_), 2.80 (t, *J* = 6.5, 2H, CH_2_NMe_2_), 4.30 (t, *J* = 6.5, 2H, CH_2_N), 7.10 (dd, *J* = 8.5, 1.8, 1H, CH_benz_), 7.55 (t, *J* = 7.5, 2H, 2CH_Ph_), 7.60 (t, *J* = 7.4, 1H, CH_Ph_), 7.77 (d, *J* = 8.5, 1H, CH_benz_), 7.94 (d, *J* = 7.4, 2H, 2CH_Ph_), 8.02 (s, 1H, CH_benz_), 8.05 (br s, 1H, NH), 8.40 (br s, 1H, CH_benz_); ^13^C NMR (75 MHz, CDCl_3_) δ 43.1 (CH_2_), 45.6 (2CH_3_), 58.4 (CH_2_), 101.6, 115.2, 120.5 (3CH), 127.0 (2CH), 128.9 (2CH), 131.9 (CH), 133.6, 134.1, 135.0, 140.6 (4C), 143.9 (CH), 165.8 (C); MS (ESI) 309.8 [M+H]^+^; Anal. (C_18_H_20_N_4_O) C, H, N.

***N*-{1-[2-(Dimethylamino)ethyl]-1*H*-benzimidazol-7-yl}benzamide (S10).** Obtained from 7-aminobenzimidazole **30** (180 mg, 0.88 mmol) in 80% yield (217 mg). Chromatography: dichloromethane/EtOH from 9.5:0.5 to 9:1; mp 98-101 ºC; IR (ATR) ν 3164, 1655, 1587, 1506, 1435; ^1^H NMR (300 MHz, CDCl_3_) δ 2.04 (s, 6H, 2CH_3_), 2.77 (t, *J* = 5.0, 2H, CH_2_NMe_2_), 4.44 (t, *J* = 5.0, 2H, CH_2_N), 7.28 (t, *J* = 7.9, 1H, CH_benz_), 7.48-7.57 (m, 3H, 3CH_Ph_), 7.66 (d, *J* = 7.6, 2H, 2CH_benz_), 7.81 (s, 1H, CH_benz_), 7.92 (d, *J* = 6.9, 2H, 2CH_Ph_), 11.04 (br s, 1H, NH); ^13^C NMR (75 MHz, CDCl_3_) δ 45.8 (CH_2_), 47.0 (2CH_3_), 60.6 (CH_2_), 118.1, 121.0, 122.6 (3CH), 122.9 (C), 127.7 (2CH), 128.6 (2CH), 131.7 (CH), 135.6, 136.1 (2C), 143.8 (CH), 145.7, 165.8 (2C); MS (ESI) 309.8 [M+H]^+^; Anal. (C_18_H_20_N_4_O) C, H, N.

***N*-{1-[2-(Dimethylamino)ethyl]-1*H*-benzimidazol-7-yl}benzenesulfonamide (S11).** Obtained from 7-aminobenzimidazole **30** (140 mg, 0.68 mmol) and benzenesulfonyl chloride (170 µL, 1.3 mmol) using general procedure A for the synthesis of final compounds in 73% yield (171 mg). Chromatography: dichloromethane/EtOH from 9.5:0.5 to 9:1; mp 142-145 ºC; IR (ATR) ν 3120, 1591, 1498, 1468, 1328, 1162; ^1^H NMR (300 MHz, CDCl_3_) δ 2.43 (s, 6H, 2CH_3_), 2.84 (m, 2H, CH_2_NMe_2_), 4.21 (m, 2H, CH_2_N), 7.13 (t, *J* = 7.9, 1H, CH_benz_), 7.20 (d, *J* = 7.9, 1H, CH_benz_), 7.44 (t, *J* = 7.8, 2H, 2CH_Ph_), 7.54 (t, *J* = 7.4, 1H, CH_Ph_), 7.63 (d, *J* = 8.0, 1H, CH_benz_), 7.73 (s, 1H, CH_benz_), 7.77 (d, *J* = 4, 2H, 2CH_Ph_); ^13^C NMR (75 MHz, CDCl_3_) δ 44.8 (CH_2_), 46.0 (2CH_3_), 60.3 (CH_2_), 118.0, 119.7 (2CH), 122.4 (C), 122.8 (CH), 126.7 (2CH), 129.1 (C), 129.3 (2CH), 132.5 (CH), 141.2 (C), 144.2 (CH), 146.0 (C); MS (ESI) 345.1 [M+H]^+^; Anal. (C_17_H_20_N_4_O_2_S) C, H, N, S.

**2. Synthesis and Characterization Data of Compounds 20-22, 30**

***N*,*N*-Dimethyl-2-(4-nitro-1*H*-benzimidazol-1-yl)ethanamine (23) and *N*,*N*-dimethyl-2-(7-nitro-1*H*-benzimidazol-1-yl)ethanamine (24).** To a suspension of 4-nitro-1*H*-benzimidazole (500 mg, 3.07 mmol) and 2-chloro-*N*,*N*-dimethylethylamine hydrochloride (670 mg, 4.65 mmol) in anhydrous dimethylformamide (DMF, 15 mL), K_2_CO_3_ (2.13 g, 15.4 mmol) and NaI (691 mg, 4.61 mmol) were added under an argon atmosphere. The reaction mixture was stirred at 60 ºC for 15 h and once at room temperature, was filtered and the solvent was evaporated under reduced pressure. The residue was resuspended in water and extracted with EtOAc (3 x 30 mL). The combined organic extracts were washed with brine, dried (MgSO_4_), filtered and evaporated. The residue was purified by flash chromatography (EtOAc to EtOAc/MeOH 9.5:0.5) to afford the title compounds **23** (239 mg, 33%) and **24** (217 mg, 30%) as oils.

**23**: IR (ATR) ν 2945, 1576, 1520, 1462; ^1^H NMR (300 MHz, CDCl_3_) δ 2.28 (s, 6H, 2CH_3_), 2.72 (t, *J* = 6.2, 2H, CH_2_NMe_2_), 4.30 (t, *J* = 6.2, 2H, CH_2_N), 7.38 (t, *J* = 8.1, 1H, CH_benz_), 7.73 (dd, *J* = 8.1, 0.7, 1H, CH_benz_), 8.11 (d, *J* = 8.1, 1H, CH_benz_), 8.24 (s, 1H, CH_benz_); ^13^C NMR (75 MHz, CDCl_3_) δ 43.7 (CH_2_), 45.5 (2CH_3_), 58.4 (CH_2_), 116.2, 119.3, 122.0 (3CH), 136.7, 137.2, 139.4 (3C), 146.8 (CH).

**24:** IR (ATR) ν 2946, 1570, 1525, 1462; ^1^H NMR (300 MHz, CDCl_3_) δ 2.16 (s, 6H, 2CH_3_), 2.55 (t, *J* = 6.3, 2H, CH_2_NMe_2_), 4.45 (t, *J* = 6.3, 2H, CH_2_N), 7.27 (t, *J* = 8.1, 1H, CH_benz_), 7.96 (d, *J* = 8.1, 1H, CH_benz_), 8.00 (d, *J* = 8.1, 1H, CH_benz_), 8.04 (s, 1H, CH_benz_); ^13^C NMR (75 MHz, CDCl_3_) δ 45.6 (CH_2_), 46.7 (2CH_3_), 59.7 (CH_2_), 120.9, 121.3 (2CH), 126.0 (C), 127.1 (CH), 136.5 (C), 147.7 (CH), 147.8 (C).

**General Procedure for the Synthesis of 25 and 27.** To a suspension of K_2_CO_3_ (1.8 equiv) in anhydrous DMF (0.7 mL/mmol), the corresponding fluoronitroaniline derivative (1 equiv) and *N,N*-dimethylethylenediamine (0.9 equiv) were added under an argon atmosphere. The reaction mixture was stirred at 90 ºC for 12 h. Once at room temperature, the mixture was filtered, washing with EtOAc, and the solvents were evaporated under reduced pressure. The residue was purified by flash chromatography to afford the title anilines as oils.

***N*^1^-[2-(Dimethylamino)ethyl]-4-nitrobenzene-1,2-diamine (25).** Obtained from 2-fluoro-5-nitroaniline (1.00 g, 6.41 mmol) and *N,N*-dimethylethylenediamine (0.62 mL, 5.76 mmol) in 78% yield (1.12 g). Chromatography: dichloromethane/EtOH 9:1; IR (ATR) ν 3350, 2926, 1618, 1568, 1507, 1466; ^1^H NMR (300 MHz, CDCl_3_) δ 2.33 (s, 6H, 2CH_3_), 2.68 (t, *J* = 6.0, 2H, CH_2_NMe_2_), 3.26 (t, *J* = 6.3, 2H, CH_2_NH), 3.30 (br s, 1H, NH), 5.00 (br s, 2H, NH_2_), 6.54 (d, *J* = 8.7, 1H, CH_Ph_), 7.63 (d, *J* = 2.7, 1H, CH_Ph_), 7.84 (dd, *J* = 8.7, 2.4, 1H, CH_Ph_); ^13^C NMR (75 MHz, CDCl_3_) δ 40.6 (CH_2_), 45.1 (2CH_3_), 57.4 (CH_2_), 108.3, 111.6, 119.0 (3CH), 132.4, 138.2, 144.5 (3C).

***N*^3^-[2-(Dimethylamino)ethyl]-4-nitrobenzene-1,3-diamine (27).** Obtained from 3-fluoro-4-nitroaniline (640 mg, 4.10 mmol) and *N,N*-dimethylethylenediamine (0.40 mL, 3.69 mmol) in 60% yield (550 mg). Chromatography: dichloromethane/EtOH from 9.5:0.5 to 9:1; IR (ATR) ν 3444, 3352, 3231, 1621, 1568, 1507, 1465; ^1^H NMR (300 MHz, CDCl_3_) δ 2.34 (s, 6H, 2CH_3_), 2.66 (t, *J* = 6.5, 2H, CH_2_NMe_2_), 3.32 (q, *J* = 6.4, 2H, CH_2_NH), 4.31 (br s, 2H, NH_2_), 5.88 (d, *J* = 2.3, 1H, CH_Ph_), 6.00 (dd, *J* = 9.2, 2.3, 1H, CH_Ph_), 8.05 (br s, 1H, NH), 8.09 (d, *J* = 9.2, 1H, CH_Ph_); ^13^C NMR (75 MHz, CDCl_3_) δ 40.9 (CH_2_), 45.4 (2CH_3_), 57.5 (CH_2_), 94.1, 104.9 (2CH), 129.8 (CH, C), 148.0, 153.6 (2C).

***N*,*N*-Dimethyl-2-(5-nitro-1*H*-benzimidazol-1-yl)ethanamine (26).** To a suspension of benzene-1,2-diamine **25** (500 mg, 2.23 mmol) in water (2.5 mL), formic acid (0.24 mL, 6.47 mmol) was added and the mixture was heated at reflux for 3 h. Once at room temperature, the reaction was poured on ice and 1 M KOH was added until pH 9-10. The aqueous solution was extracted with EtOAc (3 x 50 mL), the organic layers were dried (Na_2_SO_4_), filtered and evaporated to afford benzimidazole **26** as an oil in 92% (480 mg), which was used in the next step without further purification. IR (ATR) ν 1617, 1587, 1508, 1461; ^1^H NMR (300 MHz, CDCl_3_) δ 2.31 (s, 6H, 2CH_3_), 2.75 (t, *J* = 6.2, 2H, CH_2_NMe_2_), 4.29 (t, *J* = 6.3, 2H, CH_2_N), 7.47 (d, *J* = 8.9, 1H, CH_benz_), 8.21 (s, 1H, CH_benz_), 8.27 (dd, *J* = 8.9, 2.1, 1H, CH_benz_), 8.74 (d, *J* = 2.1, 1H, CH_benz_); ^13^C NMR (75 MHz, CDCl_3_) δ 43.7 (CH_2_), 45.6 (2CH_3_), 58.4 (CH_2_), 110.0, 117.7, 119.3 (3CH), 138.3, 143.5, 144.3 (3C), 147.0 (CH).

**General Procedure for the Synthesis of 20, 21, and 30.** To a solution of nitrobenzimidazole **23, 24** or **26** (4.27 mmol) in anhydrous MeOH (35 mL), 10% Pd(C) (86 mg) was added under an argon atmosphere and the reaction mixture was hydrogenated in a Parr hydrogenation apparatus (50 psi) at room temperature for 15 h. Then, the mixture was filtered through a pad of celite and washed with MeOH. The solvent was evaporated under reduced pressure to afford the title compounds as oils in quantitative yield, which were used in the next step without further purification.

**1-[2-(Dimethylamino)ethyl]-1*H*-benzimidazol-4-amine (20).** IR (ATR) ν 3350, 3213, 1671, 1618, 1496, 1456; ^1^H NMR (300 MHz, CDCl_3_) δ 2.29 (s, 6H, 2CH_3_), 2.70 (t, *J* = 6.9, 2H, CH_2_NMe_2_), 4.18 (t, *J* = 6.9, 2H, CH_2_N), 4.20 (br s, 2H, NH_2_), 6.53 (d, *J* = 7.6, 1H, CH_benz_), 6.78 (d, *J* = 8.0, 1H, CH_benz_), 7.10 (t, *J* = 7.9, 1H, CH_benz_), 7.84 (s, 1H, CH_benz_); ^13^C NMR (75 MHz, CDCl_3_) δ 43.3 (CH_2_), 45.6 (2CH_3_), 58.5 (CH_2_), 99.2, 105.8, 124.0 (3CH), 132.8, 134.6, 139.1 (3C), 141.1 (CH).

**1-[2-(Dimethylamino)ethyl]-1*H*-benzimidazol-5-amine (21).** IR (ATR) ν 3336, 3213, 1629, 1498, 1457; ^1^H NMR (300 MHz, CDCl_3_) δ 2.31 (s, 6H, 2CH_3_), 2.71 (t, *J* = 6.8, 2H, CH_2_NMe_2_), 4.18 (t, *J* = 6.8, 2H, CH_2_N), 6.74 (dd, *J* = 8.5, 2.0, 1H, CH_benz_), 7.10 (d, *J* = 2.0, 1H, CH_benz_), 7.18 (d, *J* = 8.5, 1H, CH_benz_), 7.86 (s, 1H, CH_benz_); ^13^C NMR (75 MHz, CDCl_3_) δ 43.4 (CH_2_), 45.6 (2CH_3_), 58.6 (CH_2_), 105.3, 109.8, 113.1 (3CH), 127.9 (C), 143.2 (CH), 142.0, 144.9 (2C).

**1-[2-(Dimethylamino)ethyl]-1*H*-benzimidazol-7-amine (30).** IR (ATR) ν 3353, 3212, 1675, 1617, 1497, 1457; ^1^H NMR (300 MHz, CDCl_3_) δ 2.28 (s, 6H, 2CH_3_), 2.81 (t, *J* = 5.9, 2H, CH_2_NMe_2_), 4.50 (t, *J* = 5.9, 2H, CH_2_N), 6.59 (d, *J* = 7.8, 1H, CH_benz_), 7.07 (d, *J* = 7.5, 1H, CH_benz_), 7.29 (t, *J* = 7.9, 1H, CH_benz_), 7.78 (s, 1H, CH_benz_); ^13^C NMR (75 MHz, CDCl_3_) δ 45.9 (CH_2_), 46.5 (2CH_3_), 61.5 (CH_2_), 111.1, 112.0, 123.3 (3CH), 124.8, 133.9 (2C), 143.9 (CH), 146.1 (C).

**1-[2-(Dimethylamino)ethyl]-1*H*-benzimidazol-6-amine (22).** To a solution of **27** (605 mg, 2.7 mmol) in MeOH (5 mL), 10% Pd(C) (108 mg) was added and the mixture was stirred for 30 min at room temperature. Then, formic acid (18 mL, 0.48 mol) was added and the reaction was stirred at reflux for 20 h. Once at room temperature, the solvent was evaporated at reduced pressure and the residue was treated with EtOAc and 1 M NaOH until pH 9-10. The organic layer was washed with brine, dried (Na_2_SO_4_), filtered and evaporated. The crude was purified by column chromatography (dichloromethane/EtOH/NH_3_ 8:2:0.1) to afford {1-[2-(dimethylamino)ethyl]-1*H*-benzimidazol-6-yl}formamide as an oil in 69% yield (430 mg). IR (ATR) ν 3247, 1675, 1623, 1602, 1550, 1498, 1458; ^1^H NMR (300 MHz, CDCl_3_, mixture of rotamers) δ 2.31 (s, 6H, 2CH_3_), 2.74 (t, *J* = 6.5, 2H, CH_2_NMe_2_), 4.22-4.27 (m, 2H, CH_2_N), 4.81 (br s, 1H, NH), 6.97 (dd, *J* = 8.6, 2.0, 1/2H, CH_benz_), 7.02 (dd, *J* = 8.6, 2.0, 1/2H, CH_benz_), 7.13 (d, *J* = 1.9, 1/2H, CH_benz_), 7.72 (d, *J* = 8.6, 1/2H, CH_benz_), 7.78 (d, *J* = 8.6, 1/2H, CH_benz_), 8.00 (s, 1/2H, CHO), 8.02 (s, 1/2H, CHO), 8.22 (d, *J* = 1.9, 1/2H, CH_benz_), 8.44 (s, 1/2H, CH_benz_), 8.45 (s, 1/2H, CH_benz_); ^13^C NMR (75 MHz, CDCl_3_, mixture of rotamers) δ 43.2, 43.4 (CH_2_), 45.6, 45.7 (2CH_3_), 58.5, 58.6 (CH_2_), 101.0, 102.0, 114.9, 115.6, 120.5, 121.5 (3CH), 132.4, 132.9, 134.1, 140.7, 141.7 (3C), 144.0, 144.2 (CH), 159.3, 163.2 (CH).

To a solution of {1-[2-(dimethylamino)ethyl]-1*H*-benzimidazol-6-yl}formamide (420 mg, 1.8 mmol) in THF (11 mL), 4 M H_2_SO_4_ (10.8 mL) was added and the reaction mixture was stirred at 50 ºC for 1 h under an argon atmosphere. Once at room temperature, the solvent was evaporated under reduced pressure and residue was basified with 2 M NaOH and extracted with dichloromethane (4 x 25 mL). The organic layers were washed with brine, dried (Na_2_SO_4_), filtered and evaporated to afford aminobenzimidazole **22** as an oil (365 mg, 99%), which was used in the next step without further purification. IR (ATR) ν 3335, 2926, 1617, 1497, 1458; ^1^H NMR (300 MHz, CDCl_3_) δ 2.30 (s, 6H, 2CH_3_), 2.69 (t, *J* = 6.9, 2H, CH_2_NMe_2_), 3.65 (br s, 2H, NH_2_), 4.13 (t, *J* = 6.9, 2H, CH_2_N), 6.65-6.70 (m, 2H, 2CH_benz_), 7.56 (d, *J* = 8.4, 1H, CH_benz_), 7.78 (s, 1H, CH_benz_); ^13^C NMR (75 MHz, CDCl_3_) δ 43.5 (CH_2_), 46.1 (2CH_3_), 58.8 (CH_2_), 94.9, 112.6, 121.3 (3CH), 135.3, 137.8, 143.3 (3C), 142.2 (CH).

**3. Characterization Data of Final Compounds 2−6, 8−19**

***N*-{1-[2-(Dimethylamino)ethyl]-1*H*-benzimidazol-4-yl}benzenesulfonamide (2).** Obtained from 4-aminobenzimidazole **20** (40 mg, 0.20 mmol) and benzenesulfonyl chloride (25 µL, 0.20 mmol) using general procedure A in 90% yield (62 mg). Chromatography: dichloromethane/EtOH from 9.5:0.5 to 9:1; mp 121-123 ºC; IR (ATR) ν 3047, 1597, 1497, 1461, 1334, 1162; ^1^H NMR (300 MHz, CDCl_3_) δ 2.29 (s, 6H, 2CH_3_), 2.71 (t, *J* = 6.6, 2H, CH_2_NMe_2_), 4.20 (t, *J* = 6.6, 2H, CH_2_N), 7.10 (d, *J* = 8.1, 1H, CH_benz_), 7.22 (t, *J* = 8.0, 1H, CH_benz_), 7.37 (t, *J* = 8.1, 2H, 2CH_Ph_), 7.46-7.47 (m, 2H, CH_Ph_, CH_benz_), 7.89 (d, *J* = 7.4, 2H, 2CH_Ph_), 7.93 (s, 1H, CH_benz_); ^13^C NMR (75 MHz, CDCl_3_) δ 43.4 (CH_2_), 45.5 (2CH_3_), 58.4 (CH_2_), 105.4, 110.8, 123.6 (3CH), 127.2 (2CH), 128.9 (2CH), 132.7 (CH), 128.7, 134.2, 134.7, 139.6 (4C), 142.7 (CH); MS (ESI) 345.1 [M+H]^+^; Anal. (C_17_H_20_N_4_O_2_S) C, H, N, S.

***N*-{1-[2-(Dimethylamino)ethyl]-1*H*-benzimidazol-5-yl}benzenesulfonamide (3).** Obtained from 5-aminobenzimidazole **21** (82 mg, 0.40 mmol) and benzenesulfonyl chloride (51 µL, 0.40 mmol) using general procedure A in 71% yield (98 mg). Chromatography: dichloromethane/EtOH from 9.5:0.5 to 9:1; mp 178-180 ºC; IR (ATR) ν 3067, 1497, 1460, 1328, 1160; ^1^H NMR (300 MHz, CDCl_3_) δ 2.29 (s, 6H, 2CH_3_), 2.71 (t, *J* = 6.6, 2H, CH_2_NMe_2_), 4.21 (t, *J* = 6.6, 2H, CH_2_N), 6.65 (br s, 1H, NH), 7.22 (dd, *J* = 8.7, 1.8, 1H, CH_benz_), 7.32 (d, *J* = 8.8, 1H, CH_benz_), 7.33 (d, *J* = 1.8, 1H, CH_benz_), 7.42 (t, *J* = 7.8, 2H, 2CH_Ph_), 7.52 (t, *J* = 7.5, 1H, CH_Ph_), 7.73 (d, *J* = 8.1, 2H, 2CH_Ph_), 8.00 (s, 1H, CH_benz_); ^13^C NMR (75 MHz, CDCl_3_) δ 43.4 (CH_2_), 45.6 (2CH_3_), 58.5 (CH_2_), 110.0, 115.7, 120.4 (3CH), 127.3 (2CH), 129.0 (2CH), 130.7, 132.5 (2C), 132.9 (CH), 139.0, 143.8 (2C), 144.5 (CH); MS (ESI) 345.8 [M+H]^+^; Anal. (C_17_H_20_N_4_O_2_S) C, H, N, S.

***N*-{1-[2-(Dimethylamino)ethyl]-1*H*-benzimidazol-6-yl}benzenesulfonamide (4).** Obtained from 6-aminobenzimidazole **22** (96 mg, 0.47 mmol) and benzenesulfonyl chloride (62 µL, 0.49 mmol) using general procedure A in 25% yield (41 mg). Chromatography: dichloromethane to dichloromethane/EtOH 9:1; mp 138-140 ºC; IR (ATR) ν 3249, 3063, 1494, 1466, 1314, 1159; ^1^H NMR (300 MHz, CDCl_3_) δ 2.29 (s, 6H, 2CH_3_), 2.69 (t, *J* = 6.4, 2H, CH_2_NMe_2_), 4.20 (t, *J* = 6.4, 2H, CH_2_N), 6.75 (dd, *J* = 8.5, 1.9, 1H, CH_benz_), 7.36-7.41 (m, 3H, CH_benz_, 2CH_Ph_), 7.51 (t, *J* = 7.4, 1H, CH_Ph_), 7.58 (d, *J* = 8.6, 1H, CH_benz_), 7.79 (d, *J* = 7.4, 2H, 2CH_Ph_), 7.99 (s, 1H, CH_benz_); ^13^C NMR (75 MHz, CDCl_3_) δ 43.5 (CH_2_), 45.9 (2CH_3_), 58.7 (CH_2_), 105.6, 118.7, 121.2 (3CH), 127.6 (2CH), 129.3 (2CH), 133.3 (CH), 131.9, 134.6, 139.3, 142.4 (4C), 144.7 (CH); MS (ESI) 345.1 [M+H]^+^; Anal. (C_17_H_20_N_4_O_2_S) C, H, N, S.

**5-Chloro-*N*-{1-[2-(dimethylamino)ethyl]-1*H*-benzimidazol-4-yl}naphthalene-2-sulfonamide (5).** Obtained from 4-aminobenzimidazole **20** (100 mg, 0.49 mmol) and 5-chloronaphthalene-2-sulfonyl chloride (134 mg, 0.51 mmol) using general procedure A in 50% yield (104 mg). Chromatography: dichloromethane/EtOH 9.5:0.5; mp 76-78 ºC; IR (ATR) ν 3093, 1743, 1657, 1594, 1567, 1537, 1500, 1466, 1330, 1159; ^1^H NMR (300 MHz, CD_3_OD) δ 2.08 (s, 6H, 2CH_3_), 2.56 (t, *J* = 6.6, 2H, CH_2_NMe_2_), 4.16 (t, *J* = 6.6, 2H, CH_2_N), 7.11 (d, *J* = 7.7, 1H, CH_benz_), 7.16 (t, *J* = 7.2, 1H, CH_benz_), 7.29 (d, *J* = 7.5, 1H, CH_benz_), 7.41 (t, *J* = 8.0, 1H, CH_naph_), 7.60 (d, *J* = 6.9, 1H, CH_naph_), 7.78 (d, *J* = 8.0, 1H, CH_naph_), 7.83 (d, *J* = 9.0, 1H, CH_naph_), 7.91 (s, 1H, CH_benz_), 8.10 (d, *J* = 8.9, 1H, CH_naph_), 8.33 (s, 1H, CH_naph_); ^13^C NMR (75 MHz, CD_3_OD) δ 43.9 (CH_2_), 45.6 (2CH_3_), 59.0 (CH_2_), 108.1, 114.9, 124.6, 125.0, 126.5, 128.7, 129.7, 130.1 (9CH), 133.1, 133.2, 133.8, 134.6, 137.3, 139.6, 144.3 (7C), 144.6 (CH); MS (ESI) 429.0 [M(^35^Cl)+H]^+^, 431.0 [M(^37^Cl)+H]^+^; Anal. (C_21_H_21_ClN_4_O_2_S) C, H, N, S.

**5-Chloro-*N*-{1-[2-(dimethylamino)ethyl]-1*H*-benzimidazol-5-yl}naphthalene-2-sulfonamide (6).** Obtained from 5-aminobenzimidazole **21** (204 mg, 1.0 mmol) and 5-chloronaphthalene-2-sulfonyl chloride (273 mg, 1.1 mmol) using general procedure A in 69% yield (296 mg). Chromatography: dichloromethane/EtOH 9.5:0.5; mp 92-95 ºC; IR (ATR) ν 3093, 1743, 1657, 1594, 1567, 1537, 1500, 1466, 1330, 1159; ^1^H NMR (300 MHz, CD_3_OD) δ 2.23 (s, 6H, 2CH_3_), 2.70 (t, *J* = 6.6, 2H, CH_2_NMe_2_), 4.29 (t, *J* = 6.6, 2H, CH_2_N), 7.11 (dd, *J* = 8.7, 1.9, 1H, CH_benz_), 7.36 (d, *J* = 1.8, 1H, CH_benz_), 7.42 (d, *J* = 8.9, 1H, CH_benz_), 7.47 (t, *J* = 8.0, 1H, CH_naph_), 7.69 (dd, *J* = 7.5, 1.0, 1H, CH_naph_), 7.82 (d, *J* = 9.0, 1H, CH_naph_), 7.85 (dd, *J* = 9.0, 1.8, 1H, CH_naph_), 8.12 (s, 1H, CH_benz_), 8.25-8.27 (m, 2H, 2CH_naph_); ^13^C NMR (75 MHz, CD_3_OD) δ 43.8 (CH_2_), 45.6 (2CH_3_), 59.1 (CH_2_), 111.7, 114.6, 120.7, 125.0, 126.6, 128.7, 129.7, 130.0, 130.1 (9CH), 132.9, 133.0, 133.2, 133.7, 134.7, 139.0, 144.2 (7C), 146.0 (CH); MS (ESI) 429.1 [M(^35^Cl)+H]^+^, 431.1 [M(^37^Cl)+H]^+^; Anal. (C_21_H_21_ClN_4_O_2_S) C, H, N, S.

**5-Chloro-*N*-{1-[2-(dimethylamino)ethyl]-1*H*-benzimidazol-4-yl}-3-methyl-1-benzothiophene-2-sulfonamide (8).** Obtained from 4-aminobenzimidazole **20** (100 mg, 0.49 mmol) and 5-chloro-3-methyl-1-benzothiophene-2-sulfonyl chloride (143 mg, 0.51 mmol) using general procedure A in 31% yield (69 mg). Chromatography: dichloromethane/EtOH 9.5:0.5; mp 90-93 ºC; IR (ATR) ν 3224, 3101, 1738, 1664, 1592, 1499, 1464, 1340, 1159; ^1^H NMR (300 MHz, CD_3_OD) δ 2.22 (s, 6H, 2NCH_3_), 2.46 (s, 3H, CH_3_), 2.70 (t, *J* = 6.6, 2H, CH_2_NMe_2_), 4.30 (t, *J* = 6.6, 2H, CH_2_N), 7.23 (t, *J* = 8.0, 1H, CH_benz_), 7.32-7.38 (m, 3H, 2CH_benz_, CH_bzthio_), 7.74-7.76 (m, 2H, 2CH_bzthio_), 8.04 (s, 1H, CH_benz_); ^13^C NMR (75 MHz, CD_3_OD) δ 12.3 (CH_3_), 43.5 (CH_2_), 45.5 (2CH_3_), 58.4 (CH_2_), 106.1, 112.1, 123.2, 123.5, 123.6, 127.5 (6CH), 128.1, 131.2, 134.5, 135.2, 136.7, 136.8, 137.8, 140.7 (8C), 143.4 (CH); MS (ESI) 449.0 [M(^35^Cl)+H]^+^, 451.0 [M(^37^Cl)+H]^+^; Anal. (C_20_H_21_ClN_4_O_2_S_2_) C, H, N, S.

**5-Chloro-*N*-{1-[2-(dimethylamino)ethyl]-1*H*-benzimidazol-5-yl}-3-methyl-1-benzothiophene-2-sulfonamide (9).** Obtained from 5-aminobenzimidazole **21** (67 mg, 0.33 mmol) and 5-chloro-3-methyl-1-benzothiophene-2-sulfonyl chloride (97 mg, 0.35 mmol) using general procedure A in 33% yield (48 mg). Chromatography: dichloromethane/EtOH 9.5:0.5; mp 90-93 ºC; IR (ATR) ν 3075, 1497, 1465, 1330, 1156; ^1^H NMR (300 MHz, CD_3_OD) δ 2.16 (s, 6H, 2NCH_3_), 2.52 (s, 3H, CH_3_), 2.60 (t, *J* = 6.2, 2H, CH_2_NMe_2_), 4.26 (t, *J* = 6.2, 2H, CH_2_N), 7.00 (dd, *J* = 8.6, 2.0, 1H, CH_benz_), 7.37 (d, *J* = 1.9, 1H, CH_benz_), 7.51 (d, *J* = 8.6, 1H, CH_benz_), 7.55 (dd, *J* = 8.6, 2.0, 1H, CH_bzthio_), 7.98 (d, *J* = 1.9, 1H, CH_bzthio_), 8.04 (d, *J* = 8.6, 1H, CH_bzthio_), 8.18 (s, 1H, CH_benz_); ^13^C NMR (75 MHz, CD_3_OD) δ 11.8 (CH_3_), 42.1 (CH_2_), 45.0 (2CH_3_), 57.9 (CH_2_), 110.7, 113.2, 118.0, 123.4, 124.7, 127.4 (6CH), 130.4, 130.5, 131.9, 136.0, 136.6, 137.1, 140.4, 143.4 (8C), 145.3 (CH); MS (ESI) 448.9 [M(^35^Cl)+H]^+^, 450.8 [M(^37^Cl)+H]^+^; Anal. (C_20_H_21_ClN_4_O_2_S_2_) C, H, N, S.

**5-Chloro-*N*-{1-[2-(dimethylamino)ethyl]-1*H*-benzimidazol-6-yl}-3-methyl-1-benzothiophene-2-sulfonamide (10).** Obtained from 6-aminobenzimidazole **22** (101 mg, 0.49 mmol) and 5-chloro-3-methyl-1-benzothiophene-2-sulfonyl chloride (145 mg, 0.51 mmol) using general procedure A in 28% yield (61 mg). Chromatography: dichloromethane to dichloromethane/EtOH 7:3; mp 114-115 ºC; IR (ATR) ν 3045, 1466, 1343, 1155; ^1^H NMR (300 MHz, CD_3_OD) δ 2.09 (s, 6H, 2NCH_3_), 2.27 (s, 3H, CH_3_), 2.53 (t, *J* = 6.7, 2H, CH_2_NMe_2_), 4.18 (t, *J* = 6.7, 2H, CH_2_N), 6.93 (dd, *J* = 8.6, 1.9, 1H, CH_benz_), 7.26 (d, *J* = 1.8, 1H, CH_benz_), 7.35 (dd, *J* = 8.7, 2.0, 1H, CH_bzthio_), 7.42 (d, *J* = 8.7, 1H, CH_benz_), 7.71 (d, *J* = 2.4, 1H, CH_bzthio_), 7.73 (d, *J* = 8.6, 1H, CH_bzthio_), 8.03 (s, 1H, CH_benz_); ^13^C NMR (75 MHz, CD_3_OD) δ 12.2 (CH_3_), 43.8 (CH_2_), 45.6 (2CH_3_), 58.9 (CH_2_), 106.1, 120.0, 120.6, 124.4, 125.0, 128.8 (6CH), 132.6, 133.7, 134.9, 137.8, 137.9, 139.1, 142.0, 142.1 (8C), 145.9 (CH); MS (ESI) 449.0 [M(^35^Cl)+H]^+^, 451.0 [M(^37^Cl)+H]^+^; Anal. (C_20_H_21_ClN_4_O_2_S_2_) C, H, N, S.

**6-Chloro-*N*-{1-[2-(dimethylamino)ethyl]-1*H*-benzimidazol-4-yl}imidazo[2,1-*b*][1,3]thiazole-5-sulfonamide (11).** Obtained from 4-aminobenzimidazole **20** (80 mg, 0.39 mmol) and 6-chloroimidazo[2,1-*b*][1,3]thiazole-5-sulfonyl chloride (105 mg, 0.41 mmol) using general procedure A in 20% yield (33 mg). Chromatography: dichloromethane/EtOH 9.5:0.5; mp 71-74 ºC; IR (ATR) ν 3326, 3117, 1741, 1667, 1591, 1494, 1460, 1267, 1123; ^1^H NMR (300 MHz, CD_3_OD) δ 2.27 (s, 6H, 2CH_3_), 2.74 (t, *J* = 6.6, 2H, CH_2_NMe_2_), 4.33 (t, *J* = 6.6, 2H, CH_2_N), 7.20-7.40 (m, 4H, 3CH_benz_, CH_imthiaz_), 7.87 (d, *J* = 4.1, 1H, CH_imthiaz_), 8.03 (s, 1H, CH_benz_); ^13^C NMR (75 MHz, CD_3_OD) δ 43.9 (CH_2_), 45.6 (2CH_3_), 59.1 (CH_2_), 109.1, 116.2, 116.6, 121.8, 124.5 (5CH), 120.3, 133.1, 136.0, 138.2, 140.3, 151.5 (6C), 144.9 (CH); MS (ESI) 425.0 [M(^35^Cl)+H]^+^, 427.0 [M(^37^Cl)+H]^+^; Anal. (C_16_H_17_ClN_6_O_2_S_2_) C, H, N, S.

**6-Chloro-*N*-{1-[2-(dimethylamino)ethyl]-1*H*-benzimidazol-5-yl}imidazo[2,1-*b*][1,3]thiazole-5-sulfonamide (12).** Obtained from 5-aminobenzimidazole **21** (86 mg, 0.42 mmol) and 6-chloroimidazo[2,1-*b*][1,3]thiazole-5-sulfonyl chloride (113 mg, 0.44 mmol) using general procedure A in 20% yield (36 mg). Chromatography: dichloromethane/EtOH 9.5:0.5; mp 99-101 ºC; IR (ATR) ν 3117, 1742, 1542, 1500, 1462, 1248; ^1^H NMR (300 MHz, CD_3_OD) δ 2.17 (s, 6H, 2CH_3_), 2.65 (t, *J* = 6.6, 2H, CH_2_NMe_2_), 4.24 (t, *J* = 6.6, 2H, CH_2_N), 7.00 (dd, *J* = 8.7, 2.0, 1H, CH_benz_), 7.18 (d, *J* = 4.5, 1H, CH_imthiaz_), 7.32 (d, *J* = 1.8, 1H, CH_benz_), 7.38 (d, *J* = 8.7, 1H, CH_benz_), 7.68 (d, *J* = 4.5, 1H, CH_imthiaz_), 8.07 (s, 1H, CH_benz_); ^13^C NMR (75 MHz, CD_3_OD) δ 43.9 (CH_2_), 45.6 (2CH_3_), 59.1 (CH_2_), 111.9, 115.0, 116.5, 120.7, 121.5 (5CH), 120.0, 132.6, 133.4, 138.6, 144.3, 151.4 (6C), 146.3 (CH); MS (ESI) 425.1 [M(^35^Cl)+H]^+^, 427.1 [M(^37^Cl)+H]^+^; Anal. (C_16_H_17_ClN_6_O_2_S_2_) C, H, N, S.

**6-Chloro-*N*-{1-[2-(dimethylamino)ethyl]-1*H*-benzimidazol-6-yl}imidazo[2,1-*b*][1,3]thiazole-5-sulfonamide (13).** Obtained from 6-aminobenzimidazole **22** (96 mg, 0.47 mmol) and 6-chloroimidazo[2,1-*b*][1,3]thiazole-5-sulfonyl chloride (125 mg, 0.49 mmol) using general procedure B in 38% yield (76 mg). Chromatography: dichloromethane to dichloromethane/EtOH 7:3; mp 139-141 ºC; IR (ATR) ν 3117, 1596, 1459, 1355, 1144; ^1^H NMR (300 MHz, CD_3_OD) δ 2.32 (s, 6H, 2CH_3_), 2.74 (t, *J* = 6.5, 2H, CH_2_NMe_2_), 4.33 (t, *J* = 6.5, 2H, CH_2_N), 7.00 (dd, *J* = 8.5, 1.4, 1H, CH_benz_), 7.31 (d, *J* = 4.4, 1H, CH_imthiaz_), 7.37 (d, *J* = 1.2, 1H, CH_benz_), 7.52 (d, *J* = 8.6, 1H, CH_benz_), 7.81 (d, *J* = 4.4, 1H, CH_imthiaz_), 8.16 (s, 1H, CH_benz_); ^13^C NMR (75 MHz, CD_3_OD) δ 43.6 (CH_2_), 45.5 (2CH_3_), 58.9 (CH_2_), 105.8, 116.7, 119.6, 120.8, 121.4 (5CH), 119.9, 133.3, 135.0, 138.7, 142.1, 151.4 (6C), 146.3 (CH); MS (ESI) 425.1 [M(^35^Cl)+H]^+^, 427.1 [M(^37^Cl)+H]^+^; Anal. (C_16_H_17_ClN_6_O_2_S_2_) C, H, N, S.

**6-Chloro-*N*-{1-[2-(dimethylamino)ethyl]-1*H*-benzimidazol-4-yl}naphthalene-2-sulfonamide (14).** Obtained from 6-aminobenzimidazole **22** (74 mg, 0.36 mmol) and 6-chloronaphthalene-2-sulfonyl chloride (95 mg, 0.36 mmol) using general procedure A in 12% yield (16 mg). Chromatography: dichloromethane to dichloromethane/EtOH 9.5:0.5; mp 181 ºC (decomposes); IR (ATR) ν 3420, 1706, 1421, 1359, 1221; ^1^H NMR (500 MHz, CD_3_OD) δ 2.16 (s, 6H, 2CH_3_), 2.57 (t, *J* = 6.6, 2H, CH_2_NMe_2_), 4.25 (t, *J* = 6.6, 2H, CH_2_N), 6.99 (dd, *J* = 8.7, 2.0, 1H, CH_benz_), 7.28 (t, *J* = 1.8, 1H, CH_benz_), 7.47 (d, *J* = 8.6, 1H, CH_benz_), 7.53 (dd, *J* = 8.8, 2.1, 1H, CH_naph_), 7.77 (dd, *J* = 8.8, 1.8, 1H, CH_naph_), 7.90 (dd, *J* = 8.8, 2.3, 2H, 2CH_naph_), 7.95 (d, *J* = 1.8, 1H, CH_naph_), 8.10 (s, 1H, CH_benz_), 8.27 (s, 1H, CH_naph_); ^13^C NMR (125 MHz, CD_3_OD) δ 43.8 (CH_2_), 45.5 (2CH_3_), 58.9 (CH_2_), 105.6, 119.6, 120.6, 124.9, 127.8, 129.4, 129.5, 129.6 (8CH), 131.7 (C), 132.0 (CH), 134.4, 135.0, 135.8, 136.8, 138.3, 141.8 (6C), 145.7 (CH); MS (ESI) 429.1 [M(^35^Cl)+H]^+^, 431.1 [M(^37^Cl)+H]^+^; Anal. (C_21_H_21_ClN_4_O_2_S) C, H, N, S.

**5-Chloro-*N*-{1-[2-(dimethylamino)ethyl]-1*H*-benzimidazol-4-yl}naphthalene-1-sulfonamide (15).** Obtained from 6-aminobenzimidazole **22** (16 mg, 0.078 mmol) and 5-chloronaphthalene-1-sulfonyl chloride (25 mg, 0.094 mmol) using general procedure A in 40% yield (13 mg). Chromatography: dichloromethane to dichloromethane/EtOH 9:1; mp 223 ºC (decomposes); IR (ATR) ν 3483, 1707, 1628, 1591, 1564, 1501, 1463, 1358, 1222, 1140; ^1^H NMR (500 MHz, CD_3_OD) δ 2.23 (s, 6H, 2CH_3_), 2.59 (t, *J* = 6.7, 2H, CH_2_NMe_2_), 4.22 (t, *J* = 6.7, 2H, CH_2_N), 6.87 (dd, *J* = 8.6, 2.0, 1H, CH_benz_), 7.15 (d, *J* = 1.8, 1H, CH_benz_), 7.39 (d, *J* = 8.6, 1H, CH_benz_), 7.57-7.62 (m, 2H, 2CH_naph_), 7.74 (dd, *J* = 7.5, 0.7, 1H, CH_naph_), 8.07 (s, 1H, CH_benz_), 8.22 (dd, *J* = 7.4, 1.0, 1H, CH_naph_), 8.48 (d, *J* = 8.6, 1H, CH_naph_), 8.76 (d, *J* = 8.7, 1H, CH_naph_); ^13^C NMR (125 MHz, CD_3_OD) δ 43.6 (CH_2_), 45.5 (2CH_3_), 58.8 (CH_2_), 105.2, 119.2, 120.5, 125.3, 126.5, 128.6, 129.0 (7CH), 130.9 (C), 131.2, 132.3 (2CH), 132.6, 133.7, 134.1, 134.8, 136.6, 141.6 (6C), 145.6 (CH); MS (ESI) 429.1 [M(^35^Cl)+H]^+^, 431.1 [M(^37^Cl)+H]^+^; Anal. (C_21_H_21_ClN_4_O_2_S) C, H, N, S.

**5-Bromo-*N*-{1-[2-(dimethylamino)ethyl]-1*H*-benzimidazol-6-yl}naphthalene-2-sulfonamide (16).** Obtained from the 6-aminobenzimidazole **22** (87 mg, 0.43 mmol) and 5-bromonaphthalene-2-sulfonyl chloride (PCT Int. Appl. WO2013086229) (137 mg, 0.45 mmol) using general procedure A in 40% yield (81 mg). Chromatography: dichloromethane to dichloromethane/methanol, 9:1; mp 226-227 ºC; IR (ATR) ν 3353, 1640, 1555, 1461, 1334, 1157; ^1^H NMR (500 MHz, CD_3_OD) δ 2.14 (s, 6H, 2CH_3_), 2.56 (t, *J* = 6.6, 2H, CH_2_NMe_2_), 4.25 (t, *J* = 6.6, 2H, CH_2_N), 7.01 (dd, *J* = 8.6, 1.9, 1H, CH_benz_), 7.26 (d, *J* = 1.6, 1H, CH_benz_), 7.44-7.50 (m, 2H, CH_benz_, CH_napth_), 7.87 (dd, *J* = 8.8, 2.3, 1H, CH_naph_), 7.92-7.97 (m, 2H, CH_naph_), 8.10 (s, 1H, CH_benz_), 8.29 (s, 1H, CH_naph_), 8.30 (d, *J* = 6.5, 1H, CH_naph_); ^13^C NMR (125 MHz, CD_3_OD) δ 42.8 (CH_2_), 44.6 (2CH_3_), 57.9 (CH_2_), 105.7, 119.8, 120.6 (3CH), 123.4 (C), 125.2, 129.2, 129.3, 130.0, 130.4, 134.0 (6CH), 134.4, 134.5, 134.8, 134.9, 139.0, 141.8 (6C), 145.8 (CH); MS (ESI) 473.0 [M(^79^Br)+H]^+^, 475.0 [M(^81^Br)+H]^+^; Anal. (C_21_H_21_BrN_4_O_2_S) C, H, N, S.

**5-Bromo-*N*-{1-[2-(dimethylamino)ethyl]-1*H*-benzimidazol-6-yl}-3-methyl-1-benzothiophene-2-sulfonamide (17).** Obtained from 6-aminobenzimidazole **22** (100 mg, 0.49 mmol) and 5-bromo-3-methyl-1-benzothiophene-2-sulfonyl chloride (PCT Int. Appl. WO2004073606) (167 mg, 0.51 mmol) using general procedure A in 33% yield (80 mg). Chromatography: dichloromethane to dichloromethane/methanol 9:1; mp 203-204 ºC; IR (ATR) ν 3098, 1503, 1465, 1344, 1155; ^1^H NMR (300 MHz, CD_3_OD) δ 2.17 (s, 6H, 2NCH_3_), 2.36 (s, 3H, CH_3_), 2.59 (t, *J* = 6.6, 2H, CH_2_NMe_2_), 4.27 (t, *J* = 6.6, 2H, CH_2_N), 7.04 (dd, *J* = 8.6, 2.0, 1H, CH_benz_), 7.34 (d, *J* = 1.8, 1H, CH_benz_), 7.52 (d, *J* = 8.7, 1H, CH_benz_), 7.56 (dd, *J* = 8.7, 1.9, 1H, CH_bzthio_), 7.74 (d, *J* = 8.6, 1H, CH_bzthio_), 7.95 (d, *J* = 1.7, 1H, CH_bzthio_), 8.13 (s, 1H, CH_benz_); ^13^C NMR (75 MHz, CD_3_OD) δ 12.2 (CH_3_), 43.8 (CH_2_), 45.6 (2CH_3_), 58.9 (CH_2_), 106.1, 120.1 (2CH), 120.2 (C), 120.6, 125.3, 127.5, 131.5 (4CH), 133.7, 134.9 (2C), 137.7 (2C), 139.5, 142.1, 142.4 (3C), 145.8 (CH); MS (ESI) 493.1 [M(^79^Br)+H]^+^, 495.1 [M(^81^Br)+H]^+^; Anal. (C_20_H_21_BrN_4_O_2_S_2_) C, H, N, S.

***N*-{1-[2-(Dimethylamino)ethyl]-1*H*-benzimidazol-6-yl}naphthalene-2-sulfonamide (18).** Obtained from the 6-aminobenzimidazole **22** (70 mg, 0.34 mmol) and naphthalene-2-sulfonyl chloride (82 mg, 0.36 mmol) using general procedure A in 30% yield (41 mg). Chromatography: dichloromethane to dichloromethane/methanol, 9:1; mp 207-208 ºC; IR (ATR) ν 3367, 1648, 1626, 1507, 1465, 1339, 1158; ^1^H NMR (500 MHz, CD_3_OD) δ 2.16 (s, 6H, 2CH_3_), 2.60 (t, *J* = 6.6, 2H, CH_2_NMe_2_), 4.26 (t, *J* = 6.6, 2H, CH_2_N), 6.99 (dd, *J* = 8.6, 2.0, 1H, CH_benz_, 7.29 (d, *J* = 1.8, 1H, CH_benz_), 7.47 (d, *J* = 8.6, 1H, CH_benz_), 7.55-7.58 (m, 1H, CH_naph_), 7.60-7.64 (m, 1H, CH_naph_), 7.73 (dd, *J* = 8.7, 1.8, 1H, CH_naph_), 7.89-7.92 (m, 2H, CH_naph_), 7.94 (d, *J* = 8.8, 1H, CH_naph_), 8.09 (s, 1H, CH_benz_), 8.27 (d, *J* = 0.9, 1H, CH_naph_); ^13^C NMR (125 MHz, CD_3_OD) δ 43.7 (CH_2_), 45.5 (2CH_3_), 58.8 (CH_2_), 105.5, 119.6, 120.5, 123.6, 128.7, 129.0, 129.6, 129.9, 130.1, 130.2 (10CH), 133.4, 134.5, 134.9, 136.2, 137.8, 141.7 (6C), 145.6 (CH); MS (ESI) 395.2 [M+H]^+^; Anal. (C_21_H_22_N_4_O_2_S) C, H, N, S.

***N*-{1-[2-(Dimethylamino)ethyl]-1*H*-benzimidazol-6-yl}-3-methyl-1-benzothiophene-2-sulfonamide (19).** Obtained from 6-aminobenzimidazole **22** (49 mg, 0.24 mmol) and 3-methyl-1-benzothiophene-2-sulfonyl chloride (62 mg, 0.25 mmol) using general procedure A in 61% yield (60 mg). Chromatography: dichloromethane to dichloromethane/methanol 9:1; mp 175-176 ºC; IR (ATR) ν 3266, 1500, 1465, 1347, 1320, 1156; ^1^H NMR (300 MHz, CD_3_OD) δ 2.15 (s, 6H, 2NCH_3_), 2.39 (s, 3H, CH_3_), 2.59 (t, *J* = 6.6, 2H, CH_2_NMe_2_), 4.26 (t, *J* = 6.6, 2H, CH_2_N), 7.03 (dd, *J* = 8.6, 2.0, 1H, CH_benz_), 7.36 (d, *J* = 1.8, 1H, CH_benz_), 7.38-7.48 (m, 2H, CH_bzthio_), 7.51 (d, *J* = 8.6, 1H, CH_benz_), 7.76-7.82 (m, 2H, CH_bzthio_), 8.11 (s, 1H, CH_benz_); ^13^C NMR (75 MHz, CD_3_OD) δ 12.3 (CH_3_), 43.7 (CH_2_), 45.5 (2CH_3_), 58.8 (CH_2_), 105.8, 119.8, 120.6, 123.4, 124.8, 126.2, 128.5 (7CH), 133.9, 134.9, 135.5, 138.6, 140.6, 140.8, 142.0 (7C), 145.7 (CH); MS (ESI) 415.1 [M+H]^+^; Anal. (C_20_H_22_N_4_O_2_S_2_) C, H, N, S.

**4.** Elemental Analysis Data

| Compd | Molecular Formula | Calculated | | | | Found | | | |
| --- | --- | --- | --- | --- | --- | --- | --- | --- | --- |
|  |  | C | H | N | S | C | H | N | S |
| **2** | C_17_H_20_N_4_O_2_S | 59.28 | 5.85 | 16.27 | 9.31 | 59.16 | 6.22 | 15.96 | 9.19 |
| **3** | C_17_H_20_N_4_O_2_S | 59.28 | 5.85 | 16.27 | 9.31 | 59.23 | 5.93 | 16.18 | 9.17 |
| **4** | C_17_H_20_N_4_O_2_S | 59.28 | 5.85 | 16.27 | 9.31 | 58.93 | 5.89 | 16.35 | 8.97 |
| **5** | C_21_H_21_ClN_4_O_2_S | 58.80 | 4.93 | 13.06 | 7.48 | 58.45 | 4.85 | 12.96 | 7.13 |
| **6** | C_21_H_21_ClN_4_O_2_S | 58.80 | 4.93 | 13.06 | 7.48 | 58.75 | 5.02 | 12.99 | 7.41 |
| **7** | C_21_H_21_ClN_4_O_2_S | 58.80 | 4.93 | 13.06 | 7.48 | 58.71 | 5.05 | 12.97 | 7.40 |
| **8** | C_20_H_21_ClN_4_O_2_S_2_ | 53.50 | 4.71 | 12.48 | 14.28 | 53.13 | 4.79 | 12.68 | 14.03 |
| **9** | C_20_H_21_ClN_4_O_2_S_2_ | 53.50 | 4.71 | 12.48 | 14.28 | 53.15 | 4.81 | 12.75 | 14.09 |
| **10** | C_20_H_21_ClN_4_O_2_S_2_ | 53.50 | 4.71 | 12.48 | 14.28 | 53.39 | 4.82 | 12.67 | 14.32 |
| **11** | C_16_H_17_ClN_6_O_2_S_2_ | 45.22 | 4.03 | 19.78 | 15.09 | 45.13 | 4.09 | 19.47 | 15.03 |
| **12** | C_16_H_17_ClN_6_O_2_S_2_ | 45.22 | 4.03 | 19.78 | 15.09 | 45.57 | 3.95 | 19.43 | 15.28 |
| **13** | C_16_H_17_ClN_6_O_2_S_2_ | 45.22 | 4.03 | 19.78 | 15.09 | 45.49 | 4.20 | 19.46 | 14.97 |
| **14** | C_21_H_21_ClN_4_O_2_S | 58.80 | 4.93 | 13.06 | 7.48 | 58.73 | 5.31 | 12.79 | 7.10 |
| **15** | C_21_H_21_ClN_4_O_2_S | 58.80 | 4.93 | 13.06 | 7.48 | 58.47 | 5.25 | 12.77 | 7.19 |
| **16** | C_21_H_21_BrN_4_O_2_S | 53.28 | 4.47 | 11.84 | 6.77 | 53.65 | 4.83 | 11.54 | 6.27 |
| **17** | C_20_H_21_BrN_4_O_2_S_2_ | 48.68 | 4.29 | 11.35 | 13.00 | 49.01 | 4.55 | 10.95 | 12.63 |
| **18** | C_21_H_22_N_4_O_2_S | 63.94 | 5.62 | 14.20 | 8.13 | 63.64 | 5.82 | 13.80 | 7.78 |
| **19** | C_20_H_22_N_4_O_2_S_2_ | 57.95 | 5.35 | 13.52 | 15.47 | 57.71 | 5.50 | 13.16 | 15.09 |
| **S1** | C_19_H_22_N_4_O | 70.78 | 6.88 | 17.38 | -- | 70.63 | 7.02 | 17.00 | -- |
| **S2** | C_19_H_22_N_4_O | 70.78 | 6.88 | 17.38 | -- | 70.59 | 6.80 | 17.12 | -- |
| **S3** | C_22_H_28_N_4_O | 72.50 | 7.74 | 15.37 | -- | 72.26 | 7.81 | 14.99 | -- |
| **S4** | C_22_H_28_N_4_O | 72.50 | 7.74 | 15.37 | -- | 72.27 | 7.77 | 15.07 | -- |
| **S5** | C_21_H_24_N_4_O | 72.39 | 6.94 | 16.08 | -- | 72.03 | 6.99 | 15.92 | -- |
| **S6** | C_21_H_24_N_4_O | 72.39 | 6.94 | 16.08 | -- | 71.96 | 7.05 | 15.86 | -- |
| **S7** | C_18_H_20_N_4_O | 70.11 | 6.54 | 18.17 | -- | 70.48 | 6.79 | 17.89 | -- |
| **S8** | C_18_H_20_N_4_O | 70.11 | 6.54 | 18.17 | -- | 69.95 | 6.50 | 18.03 | -- |
| **S9** | C_18_H_20_N_4_O | 70.11 | 6.54 | 18.17 | -- | 70.21 | 6.73 | 17.98 | -- |
| **S10** | C_18_H_20_N_4_O | 70.11 | 6.54 | 18.17 | -- | 69.88 | 6.75 | 17.79 | -- |
| **S11** | C_17_H_20_N_4_O_2_S | 59.28 | 5.85 | 16.27 | 9.31 | 59.66 | 5.99 | 16.12 | 9.15 |

**5. Affinity Data of Compound 7 toward a Panel of Receptors**

Compound **7** was screened for binding affinity toward the following receptors: serotonin 5-HT_1A_, 5-HT_2A_, 5-HT_2B_, 5-HT_4e_, and 5-HT_7_, histamine H_3_, muscarinic acetylcholine M_1_, cannabinoid CB_2_, α_2_ adrenergic, α_7_ nicotinic, and *N*-Methyl-D-aspartate (NMDA), at Eurofins Cerep (France).

| **Receptor** | **Radioligand (nM)** | **Non specific (μM)** | **Incubation** | **Inhibition (%)***^a,b^* |
| --- | --- | --- | --- | --- |
| 5-HT_1A_ | [^3^H]-8-OH-DPAT (0.3) | 8-OH-DPAT (10) | 60 min, RT | -4 |
| 5-HT_2A_ | [^3^H]ketanserin (0.5) | ketanserin (1) | 60 min, RT | 23 |
| 5-HT_2B_ | [^3^H]mesulergine | SB206553 (10) | 60 min, RT | 16 |
| 5-HT_4e_ | [^3^H]GR 113808 (0.3) | serotonin (100) | 60 min, 37 ºC | -6 |
| 5-HT_7_ | [^3^H]LSD | serotonin (10) | 120 min, RT | -0.5 |
| H_3_ | [^3^H]N^α^-Me-histamine (1) | (*R*)-α-Me-histamine (1) | 60 min, RT | -8 |
| M_1_ | [^3^H]pirenzepine (2) | atropine (1) | 60 min, RT | -8 |
| CB_2_ | [^3^H]WIN 55212-2 (0.8) | WIN 55212-2 (5) | 120 min, 37 °C | -18 |
| α_2_ adrenergic | [^3^H]RX 821002 (0.5) | (-)-epinephrine (100) | 60 min, RT | 0 |
| α_7_ nicotinic | [^125^I]α-bungarotoxin (0.05) | α-bungarotoxin (1) | 120 min, 37 °C | -17 |
| NMDA | [^3^H]CGP 39653 (5) | L-glutamate (100) | 60 min, 4 °C | 1 |

**Supplementary Table S2.** Selectivity panel of compound **7**. *^a^* Compound **7** was tested at 1 μM; *^b^* values are the mean of one experiment performed in duplicate.

**6. Mutagenesis Assays**

**Cell cultures and transfection.** HEK-293 cells were grown in Dulbecco’s modified Eagle’s medium (Gibco, UK) supplemented with 2 mM L-glutamine, 100 μg/mL sodium pyruvate, 100 U/mL penicillin/streptomycin, minimum essential medium nonessential amino acids solution (1/100), and 10% (v/v) heat‑inactivated fetal bovine serum (Pantech, Germany) at 37 ºC in a 5% CO_2_ humidified atmosphere. For cAMP measurement experiments, 50000 cells/well were seeded in poly‑D‑lysine (Sigma-Aldrich, UK) coated white clear bottom 96 well plates (Corning, UK) and transfected with 50 ng/well of pGloSensor^TM^-22F cAMP plasmid (Promega, UK) and 100 ng/well of the corresponding 5-HT_6_R. Transfections were performed using Lipofectamine 3000 reagent (Invitrogen, UK), according to the manufacturer’s instructions.

**Expression vectors and mutagenesis.** The plasmid encoding the human 5-HT_6_ receptor cDNA was obtained from the cDNA Resource Center (#HTR0600000, [www.cdna.org](http://www.cdna.org/)). C110A, S193A and N288A mutants were designed according to the protocol described by Liu and Naismith (*BMC Biotechnol.* **2008**, *8*, 91). The F285A mutant was designed based on the QuickChange™ site-directed mutagenesis protocol. For all constructs, the PCR reaction of 50 μL contained 20 ng of template, 1 μM primer pair, 200 μM dNTPs and 1 unit of Phusion Hot Start II High-Fidelity DNA Polymerase (all reagents purchased from Thermo Scientific, UK). The PCR cycles were initiated at 98 ºC for 3 min to denature the template DNA, followed by 12 amplification cycles. Each amplification cycle consisted of 98 ºC for 1 min, an annealing gradient of 60, 65 or 70 ºC for 30 s and extension at 72 ºC for 3:45 min (20 s/kb). The PCR cycles were finished with an annealing gradient step for 1 min followed by an extension at 72 ºC for 30 min. The PCR products were treated overnight with 5 units of DpnI (Thermo Scientific, UK) at 37 ºC and 10 μL of each PCR reaction was analyzed by agarose gel electrophoresis. An aliquot of 2 μL of the above PCR products was transformed into *E. coli* DH5α competent cells by heat shock. The transformed cells were spread on a Luria‑Bertani (LB) plate containing antibiotics and incubated at 37 ºC overnight. Two colonies from each plate were picked and grown and the plasmid DNA was isolated. The mutations were verified by DNA sequencing. PCR cycling was carried out using a Veriti thermal cycler (Applied Biosystems, UK). All primers are detailed in Table S3.

| **Primers** | **Sequence** |
| --- | --- |
| C110A FW | 5’-CGCAAGCGCCTCCATCCTCAACCTC-3’ |
| C110A RV | 5’-GCGCTTGCGCACATCACGTCGAAGG-3’ |
| S193A FW | 5’-TGGCGGCCGGCCTCACCTTCTTCCTGCCC-3’ |
| S193A RV | 5’-GCCGGCCGCCACAAGGACAAAAGGCAGGC-3’ |
| F285A FW | 5'-TGACCTGGTTGCCCTTCGCTGTGGCCAACATAGTCC-3' |
| F285A RV | 5'-GGACTATGTTGGCCACAGCGAAGGGCAACCAGGTCA-3' |
| N288A FW | 5’-TGGCCGCGATAGTCCAGGCCGTGTG-3’ |
| N288A RV | 5’-TATCGCGGCCACAAAGAAGGGCAAC-3’ |

**Supplementary Table S3.** Mutagenesis primers

**cAMP measurements.** The EC_50_ of 5-HT in WT receptor and each individual mutant was determined before performing competition experiments and the actual 5-HT concentration used for each receptor was 10x its EC_50_.

|  | **cAMP release (5-HT)** | |
| --- | --- | --- |
|  | **pEC_50_** | **Efficacy** |
| **5-HT_6_R WT** | 9.5 ± 0.1 | agonist |
| **5-HT_6_R C110A** | 9 ± 0.2 | agonist |
| **5-HT_6_R S193A** | 7.6 ± 0.1 | agonist |
| **5-HT_6_R F285A** | 7.1 ± 0.1 | agonist |
| **5-HT_6_R N288A** | 9 ± 0.3 | agonist |

**Supplementary Figure S2.** EC_50_ of 5-HT in WT receptor and each individual mutant.

**7. Sequence Alignment and Binding Pocket Analysis of Serotonin Receptors**

**
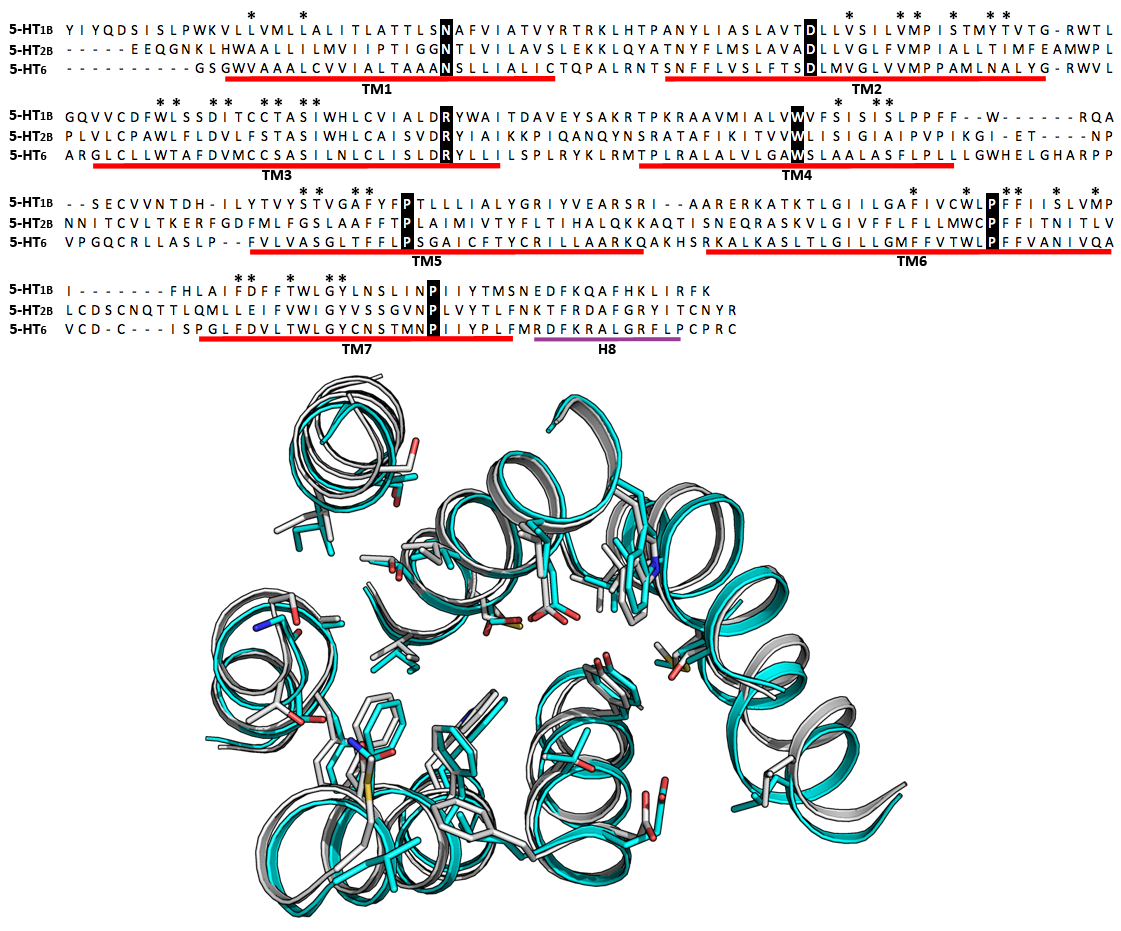
**

**Supplementary Figure S3.** Sequence alignment between crystallized 5-HT_1B_R (PDBid: 4IAR) and 5-HT_2B_R (PDBid: 4IB4) and human 5-HT_6_R (Uniprot code P50406). Transmembrane regions are underlined in red. The highly conserved residues are shown in black and amino acid residues forming the binding pocket are marked with asterisks. These residues are displayed in sticks in the lower panel, where the superposition of 5-HT_1B_R and 5-HT_2B_R structures (grey and cyan, respectively) is shown.

**8. MD Simulations of Compounds 7 and 18 in Complex with the 5-HT_6_R**


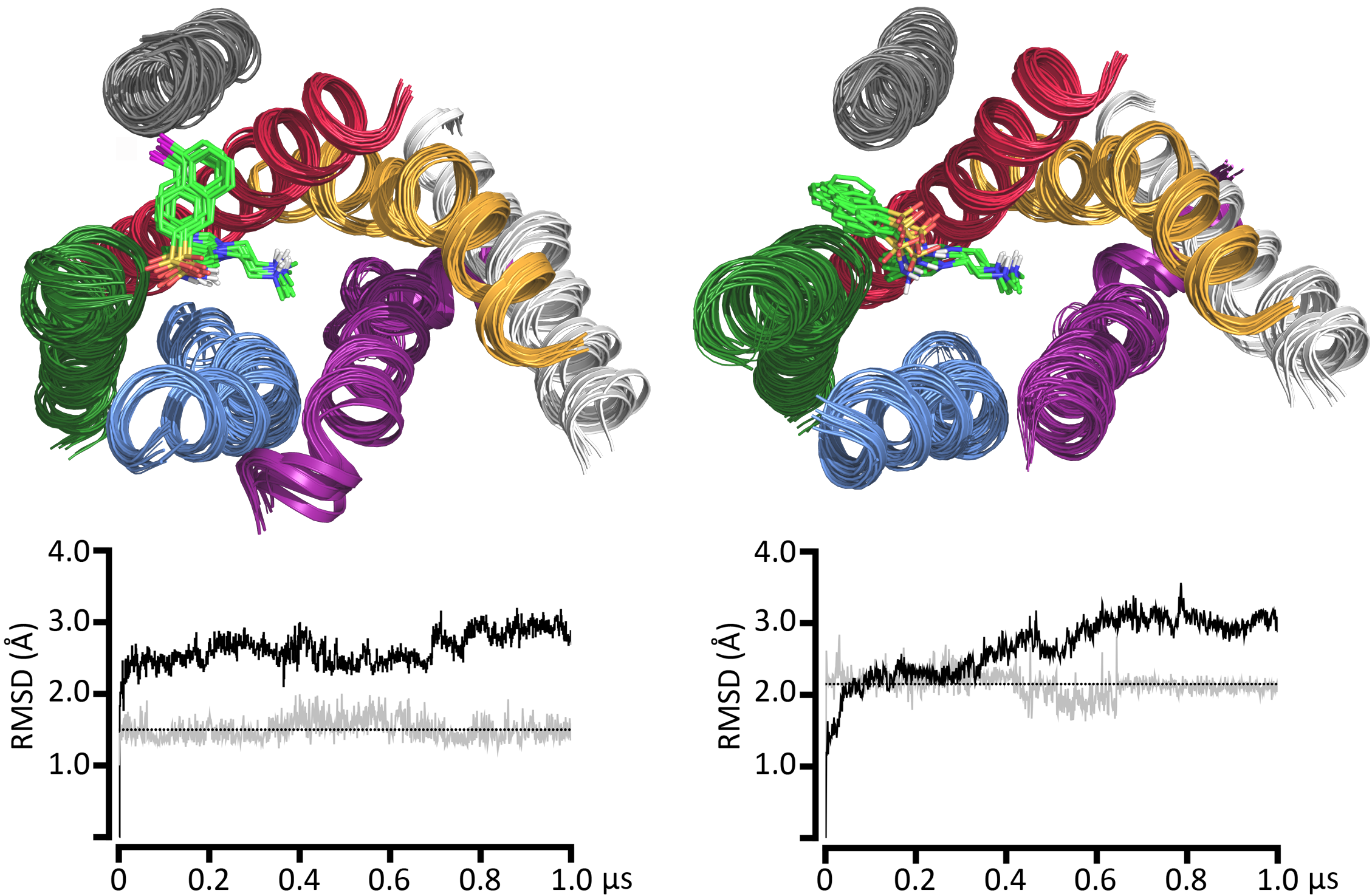


**Supplementary Figure S4.** MD simulations of the 5-HT_6_R in complex with compound **7** (left) and **18** (right). On top, representation of 10 evenly spaced snapshots extracted from 1 µs explicit membrane MD trajectories of the ligand-receptor complexes. The 5-HT_6_R ribbon structure is colored as follows: TM1 (white), TM2 (orange), TM3 (red), TM4 (grey), TM5 (green), TM6 (blue), and TM7 (purple). Loop regions of the receptor and non-polar hydrogens of the ligands (in sticks) are omitted for clarity. The graphs below show the root mean-square deviation (RMSD) of the receptor backbone atoms (black) and ligands heavy atoms (light grey) along the MD trajectories. Horizontal dashed lines indicate the average of the ligand RMSD plotted data.

**9. MD Trajectory Analysis of Compound 7 in Complex with the 5-HT_6_R**

**
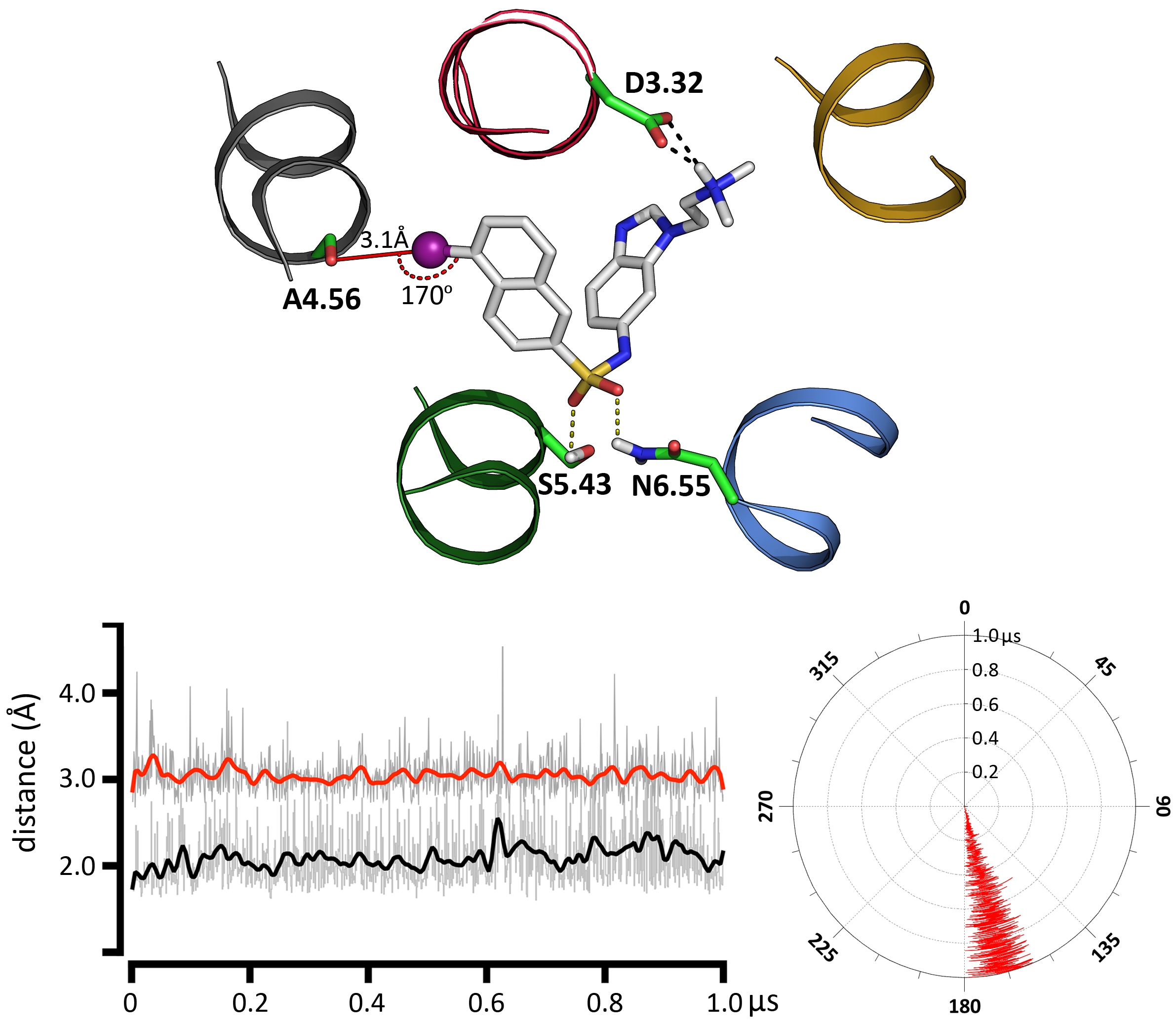
**

**Supplementary Figure S5.** Binding and dynamics of compound **7** to the 5-HT_6_R. On top, detailed view of the ligand-receptor complex. Main interactions between compound **7** and the 5-HT_6_R include a salt bridge between the protonated amine and D3.32 (black dashed lines), two hydrogen bonds between the sulfonamide group and N6.55 and S5.43 (yellow dashed lines) and the halogen bond between the chlorine substituent (purple sphere) and the backbone carbonyl of A4.56 (in red, average distance and angle are indicated). The color code of the TM helices is as in Supplementary Figure S4. The graphs below show the evolution of the distances (left panel) between the protonated amine and the carboxyl oxygen of D3.32 (black line) and between the Cl atom and the carbonyl oxygen at position 4.56 (red line) and the σ-hole angle (Cl∙∙∙O=C) of the halogen bond (right panel) obtained during the MD simulation.

**10. Molecular Systems used in Explicit Membrane MD Simulations**


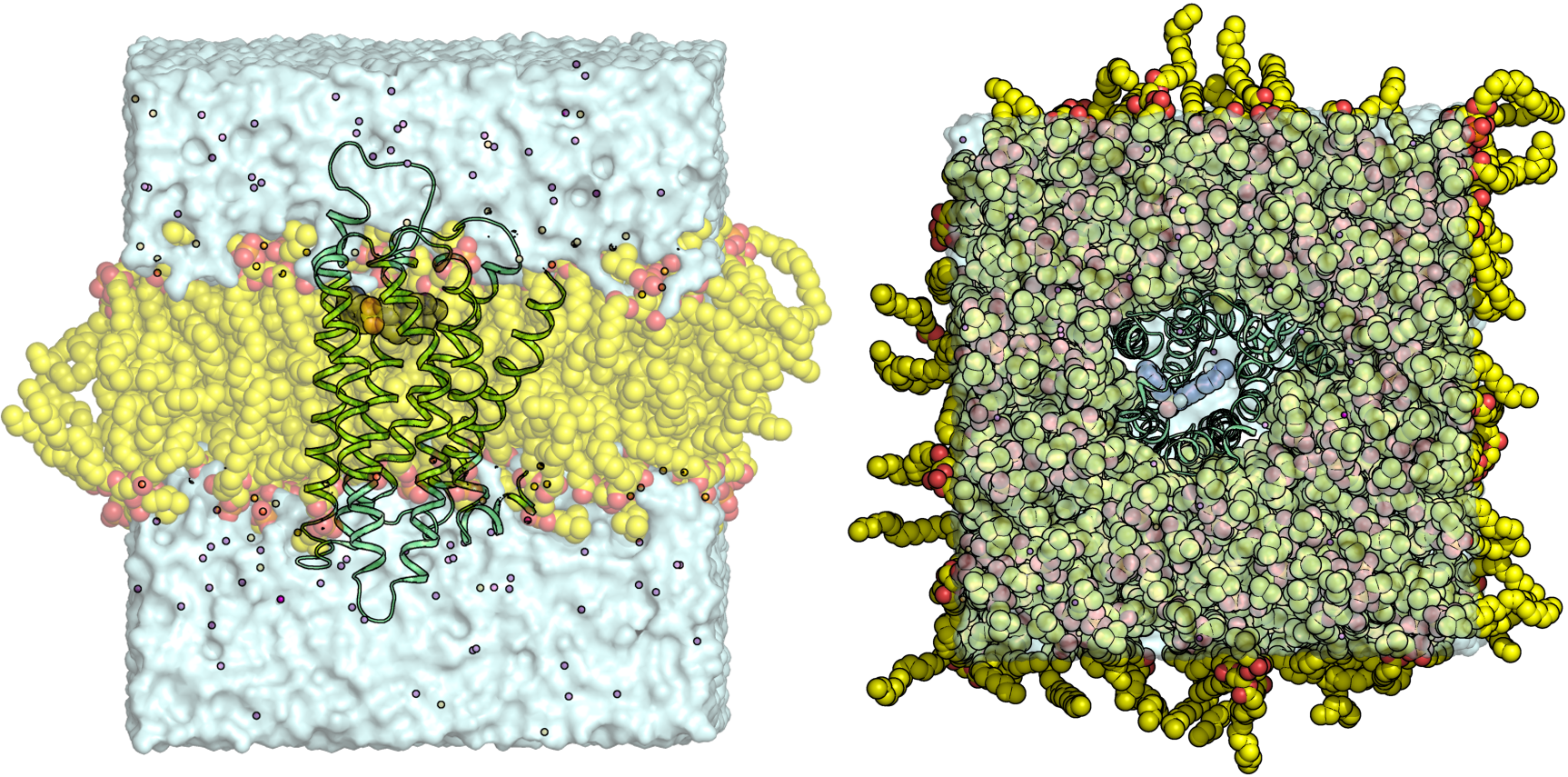


**Supplementary Figure S6.** Side and top views of the molecular system composed of the 5-HT_6_R (green ribbon) and compound **7** (vdW spheres). The ligand-receptor complex is embedded in a lipid bilayer of 200 molecules of 1-palmitoyl-2-oleoyl-*sn*-glycero-3-phosphocholine (yellow vdW spheres), 13,919 water molecules (light blue surface) and 61 Na^+^ and 76 Cl^-^ counterions (small non-bonded spheres). 1 µs MD trajectories were produced at a constant temperature of 300 K using separate v-rescale thermostats for the protein, ligand, lipids, and solvent molecules. A time step of 2 fs was used for the integration of equations of motion. All bonds and angles were kept frozen using the LINCS algorithm. Lennard-Jones interactions were computed using a cutoff of 10 Å, and the electrostatic interactions were treated using the particle mesh Ewald method. The AMBER99SB-ILDN force field was used for the protein and parameters described by Berger and co-workers for the lipids. Ligand parameters were obtained from the general Amber force field (GAFF) and HF/6-31G*-derived RESP atomic charges.
